# Simultaneous silencing of juvenile hormone metabolism genes through RNAi interrupts metamorphosis in the cotton boll weevil

**DOI:** 10.3389/fmolb.2023.1073721

**Published:** 2023-03-06

**Authors:** Daniel D. N. Vasquez, Daniele H. Pinheiro, Lays A. Teixeira, Clidia E. Moreira-Pinto, Leonardo L. P. Macedo, Alvaro L. O. Salles-Filho, Maria C. M. Silva, Isabela T. Lourenço-Tessutti, Carolina V. Morgante, Luciano P. Silva, Maria F. Grossi-de-Sa

**Affiliations:** ^1^ Embrapa Genetic Resources and Biotechnology, Brasília, Brazil; ^2^ Catholic University of Brasília, Brasília, Brazil; ^3^ Embrapa Café, Brasília, Brazil; ^4^ National Institute of Science and Technology (INCT PlantStress Biotech), Embrapa, Brasília, Brazil; ^5^ Federal University of Paraná, Curitiba, Brazil; ^6^ Embrapa SemiArid, Petrolina, Brazil

**Keywords:** *Anthonomus grandis*, juvenile diol kinase, juvenile hormone epoxide hydrolase, chitosan, polyethylenimine

## Abstract

The cotton boll weevil (CBW) (*Anthonomus grandis*) is one of the major insect pests of cotton in Brazil. Currently, CBW control is mainly achieved by insecticide application, which is costly and insufficient to ensure effective crop protection. RNA interference (RNAi) has been used in gene function analysis and the development of insect control methods. However, some insect species respond poorly to RNAi, limiting the widespread application of this approach. Therefore, nanoparticles have been explored as an option to increase RNAi efficiency in recalcitrant insects. Herein, we investigated the potential of chitosan–tripolyphosphate (CS-TPP) and polyethylenimine (PEI) nanoparticles as a dsRNA carrier system to improve RNAi efficiency in the CBW. Different formulations of the nanoparticles with dsRNAs targeting genes associated with juvenile hormone metabolism, such as *juvenile hormone diol kinase* (*JHDK*), *juvenile hormone epoxide hydrolase* (*JHEH*), and *methyl farnesoate hydrolase* (*MFE*), were tested. The formulations were delivered to CBW larvae through injection (0.05–2 µg), and the expression of the target genes was evaluated using RT-qPCR. PEI nanoparticles increased targeted gene silencing compared with naked dsRNAs (up to 80%), whereas CS-TPP-dsRNA nanoparticles decreased gene silencing (0%–20%) or led to the same level of gene silencing as the naked dsRNAs (up to 50%). We next evaluated the effects of targeting a single gene or simultaneously targeting two genes *via* the injection of naked dsRNAs or dsRNAs complexed with PEI (500 ng) on CBW survival and phenotypes. Overall, the gene expression analysis showed that the treatments with PEI targeting either a single gene or multiple genes induced greater gene silencing than naked dsRNA (∼60%). In addition, the injection of ds*JHEH/JHDK*, either naked or complexed with PEI, significantly affected CBW survival (18% for PEI nanoparticles and 47% for naked dsRNA) and metamorphosis. Phenotypic alterations, such as uncompleted pupation or malformed pupae, suggested that *JHEH* and *JHDK* are involved in developmental regulation. Moreover, CBW larvae treated with ds*JHEH*/*JHDK* + PEI (1,000 ng/g) exhibited significantly lower survival rate (55%) than those that were fed the same combination of naked dsRNAs (30%). Our findings demonstrated that PEI nanoparticles can be used as an effective tool for evaluating the biological role of target genes in the CBW as they increase the RNAi response.

## Introduction

RNA interference (RNAi) can induce a posttranscriptional gene silencing (PTGS) mechanism that is highly conserved in eukaryotes, in which double-stranded RNAs (dsRNAs) reduce the expression of target genes through the cleavage of complementary mRNAs (Hannon, 2002). RNAi has been widely used in functional genomic studies to efficiently silence insect genes and evaluate their biological role (Lenaerts et al., 2016; Yang et al., 2021). Furthermore, RNAi has been exploited to develop sustainable insect control strategies owing to its high specificity and ability to cause lethal phenotypes in insects upon the silencing of essential genes (Baum et al., 2007; Gordon and Waterhouse, 2007; Price and Gatehouse, 2008). However, the development of RNAi-based insect control strategies toward field application has remained challenging due to low RNAi efficiency in some agriculturally important insect species (Christiaens et al., 2020).

The cotton boll weevil (CBW) (*Anthonomus grandis*: Coleoptera) is considered an important insect pest of cotton in Brazil (Miranda et al., 2015). This insect may substantially reduce the yield and quality of cotton if adequate control practices are not adopted. The management of CBW is largely accomplished through the application of chemical insecticides (Oliveira-Marra et al., 2019; Rolim et al., 2021; Torres et al., 2022). However, the low cost-benefit efficiency of this control method has driven the search for alternative strategies, such as RNAi-based biopesticides.

While a moderate RNAi response is observed following the injection of small amounts of dsRNA in the CBW (Macedo et al., 2017; Firmino et al., 2020; Moreira-Pinto et al., 2021), large amounts of dsRNA are required to produce strong and persistent gene silencing that culminates in clear phenotypic effects when the dsRNA is delivered through feeding (Garcia et al., 2017). Therefore, the relatively weak RNAi response of CBW upon dsRNA treatment hampers the functional analysis of genes through the RNAi approach and the selection of potential target genes that could be used in the development of RNAi-mediated control methods.

Multiple strategies have been designed to improve the efficiency of RNAi in recalcitrant insects (Silver et al., 2021). Nanoparticles and other polymer-based complexes provide a promising approach for enhancing the RNAi response in insects by protecting dsRNA molecules against degradation and/or facilitating dsRNA cellular uptake (Pugsley et al., 2021). Chitosan (CS) is a polysaccharide composed of glucosamine and N-acetyl glucosamine residues and can be produced *via* the partial deacetylation of chitin (Bakshi et al., 2020). Because CS is non-toxic, biodegradable, and has relatively low production costs, it has been explored as a suitable system for delivering dsRNA to target insects. Several studies have examined the potential of CS in increasing the effectiveness of RNAi in different insects, such as *Anopheles gambiae* (Zhang et al., 2010; 2015), *Aedes aegypti* (Mysore et al., 2014; Das et al., 2015; Kumar et al., 2016; Dhandapani et al., 2019), *Spodoptera frugiperda* (Gurusamy et al., 2020), *Helicoverpa armigera* (Kolge et al., 2021), and *Chilo suppressalis* (Wang et al., 2020). Similarly, polyethylenimine (PEI) is a cationic polymer that has been widely used as a carrier for the delivery of siRNA and dsRNA into mammalian cells for RNAi-mediated gene silencing (Nimesh and Chandra, 2009; Sajeesh et al., 2014). Among insects, PEI has been used to transfect cell lines (Maeda et al., 2005; Ogay et al., 2006) and deliver dsRNA to *A. aegypti*, *Tribolium castaneum*, and *C. suppressalis* through carbon quantum dot (CQD)–PEI complexes (Das et al., 2015; Wang et al., 2020; Shen et al., 2022).

Juvenile hormone (JH) regulates several key biological processes in insects, which include metamorphosis, development, reproduction, and diapause (Li et al., 2019). Juvenile hormone diol kinase (JHDK), juvenile hormone epoxide hydrolase (JHEH), and methyl farnesoate hydrolase (MFE) are enzymes that play important roles in the JH metabolism pathway and function antagonistically to fine-tune the regulation of JH biosynthesis and degradation in insects. While JHEH and JHDK are JH-degrading enzymes, MFE acts in the final step of JH biosynthesis, catalyzing the conversion of methyl farnesoate (MF) to JH. Previous studies have characterized these enzymes in several insect species (Helvig et al., 2004; Tsubota et al., 2010; Lü et al., 2015; Cardoso-Júnior et al., 2017; Jiang et al., 2017; Tusun et al., 2017; Zhao et al., 2017; Guo et al., 2019; Brito et al., 2021; Li et al., 2022). However, their biological roles in the CBW remain largely unexplored.

In the present study, we evaluated different approaches with the aim of increasing the RNAi response of the CBW to better understand the biological roles of *JHDK*, *JHEH*, and *MFE* and whether these genes would be suitable targets for RNAi-mediated control methods. First, we examined the potential of CS and PEI nanoparticles in improving the RNAi silencing efficiency in CBW larvae. We found that CS and PEI nanoparticles could protect dsRNA against degradation through nucleases present in the gut juice of the CBW. However, only PEI led to increased gene silencing in the CBW. Then, we investigated the effects of silencing the *JHDK*, *JHEH*, and *MFE* genes on CBW survival and phenotypes through the injection of corresponding naked dsRNAs or dsRNAs complexed with PEI nanoparticles. In addition, the effects of simultaneously targeting two genes on CBW survival and phenotypes were evaluated. We found that ds*JHEH*/*JHDK*, ds*JHEH*/*JHDK* + PEI, and *JHEH* + PEI significantly decreased the survival of CBW compared with control treatments and compromised pupa–adult metamorphosis. Moreover, we observed increased gene silencing and mortality in the insects fed with ds*JHEH*/*JHDK* + PEI compared with those that were fed with naked ds*JHEH*/*JHDK*.

## Materials and methods

### Identification of JH degradation pathway genes and dsRNA synthesis

The full-length cDNA sequences encoding JHEH, JHDK and MFE were obtained from the CBW transcriptome. The presence of domains and motifs typically found in these proteins was confirmed through multiple alignments of the predicted proteins with homologous sequences available in GenBank. The alignment was performed using the Clustal Omega algorithm (https://www.ebi.ac.uk/Tools/msa/clustalo/). The dsRNA molecules were designed on the basis of the coding DNA sequence (CDS) of each gene using the E-RNAi web tool (https://www.dkfz.de/signaling/e-rnai3/). To mitigate possible off-target effects, a cutoff of <19 bp homology for putative sequences from honeybee (*Apis mellifera*) and fruit fly (*Drosophila melanogaster*) was used in the design of the dsRNAs. All sequence details and sources are described in Supplementary Data S1. Template DNA plasmids (pCloneEZ-NRS-Blunt-Amp) containing the partial sequences of the target genes flanked by the T7 promoter were purchased from Epoch Biolabs Inc. (Texas, USA). The vectors were transformed into chemically competent OmniMAX *E. coli*. Then, the plasmid DNA was isolated by alkaline lysis (Ehrt and Schnappinger, 2003) and used as a template for the PCR amplification of each gene fragment using specific primers (Supplementary Table S1). The PCR product was purified using a PureLink™ PCR Purification Kit according to manufacturer’s instructions (Invitrogen, Massachusetts, United States). Using 8 µL (250 ng/μL) of the purified PCR product as the template, dsRNA synthesis was performed in reactions of 20 µL using a MEGAscript T7 RNAi Kit (Invitrogen, Massachusetts, United States). The transcription reaction was run overnight and then purified according to the manufacturer’s instructions.

### Preparation and characterization of dsRNA–nanoparticle complexes

The CS-TPP-dsRNA complexes were assembled as described by Dhandapani et al. (2019) with slight modifications. Briefly, low-molecular-weight CS (85% deacetylated) (Sigma‒Aldrich, Darmstadt, Germany) was dissolved in 1% acetic acid (2 mg/mL), and sodium tripolyphosphate (TPP) (Sigma‒Aldrich, Darmstadt, Germany) was dissolved in Milli-Q ultrapure water (2.5 mg/mL). Then, 2 mL of dsRNA (2.5 mg/mL) and 2 mL of TPP (2.5 mg/mL) were added dropwise to 5 mL of CS solution under magnetic stirring, and the solution was stirred for 60 min at room temperature. The CS-TPP-dsRNA complex was prepared using a mass ratio of 5:10:1. Linear PEI 20 KDa (Invitrogen, Missouri, United States) was dissolved in Milli-Q ultrapure water (1 mg/mL). Thereafter, the dsRNA-PEI complexes were prepared by mixing 6 mL of Milli-Q ultrapure water, 2 mL of dsRNA (2.5 mg/mL), and 2 mL of PEI (1 mg/mL) under magnetic stirring for 60 min at room temperature. The dsRNA-PEI complex was prepared using a nitrogen:phosphate ratio of 6:1. Finally, both complexes were placed in an ultrasonic bath for 15 min. The final concentration of dsRNA in CS-TPP and PEI complexes was 500 ng/μL. Apart from the CS:dsRNA and PEI:dsRNA ratios mentioned above, 1:1 and 10:1 CS:dsRNA, as well as 1:1, 3:1, 10:1, and 20:1 N:P ratios (for PEI:dsRNA), were also formulated keeping the dsRNA and TPP amounts constant.

The CS-TPP-dsRNA and PEI-dsRNA complexes were characterized by dynamic light scattering (DLS) to determine their mean diameter (z-average), surface charge (zeta potential), and polydispersity (PdI). DLS measurements were performed using a Malvern Zetasizer Nano ZS (Malvern Panalytical, Worcestershire, United Kingdom) with three readings taken per sample with the following parameters: 25°C, material RI of 1.59, and dispersant (Milli-Q ultrapure water) RI and viscosity of 1.33 and 0.887, respectively. Samples for the DLS analysis were diluted to 50 ng/μL of dsRNA.

### Insect rearing

CBWs were obtained from the insect rearing platform of Embrapa Genetic Resources and Biotechnology (Brasília, DF, Brazil). The weevils were reared on an artificial diet (Supplementary Table S2) and maintained under standard rearing conditions of 28°C, 70% ± 5% relative humidity, and a 12:12 h light:dark photoperiod.

### *Ex vivo* incubation of naked dsRNA and dsRNA complexed with nanoparticles in hemolymph and gut fluids

To perform the dsRNA degradation assay, hemolymph and gut fluid were extracted from third-instar CBW larvae, as described by Cooper *et al.* (2020). Briefly, the hemolymph was extracted from 10 individuals by performing a small incision on the dorsal side of the abdomen, and the larvae were allowed to bleed out on ice-cold PARAFILM (Sigma‒Aldrich, Missouri, United States). Then, the hemolymph was collected using a micropipette and placed into a tube containing 50 mg of phenylthiourea (Sigma‒Aldrich, Missouri, United States). For gut juice extraction, 10 individuals were gently held with tweezers by the anterior part of the body. Then, a gentle pressure was applied longitudinally in the larvae to stimulate peristaltic movements across the gut. After emesis, the gut juice was collected into a tube and centrifuged at 14,000*g* for 20 min at 4°C, and the supernatant was transferred to another tube. Protein contents from the hemolymph and gut juice were determined by fluorometry using a Quibit Protein Assay Kit in a Qubit 4 Fluorometer (Invitrogen, Massachusetts, United States). Then, the extracted hemolymph and gut fluid were diluted using PBS buffer to 500 ng/μL for an *ex vivo* assay.

For the *ex vivo* assay, 2 µL (250 ng/μL) of naked ds*GFP* (400 bp) and ds*GFP* complexed with CS-TPP or PEI were mixed with 3 µL (1.5 µg) of the hemolymph or gut fluids and 15 µL of 1× PBS. The pH of PBS was adjusted according to the pH of insect fluids (pH = 7.0 for hemolymph and pH = 6.0 for gut fluid). Control treatments included these mixtures without dsRNA or body fluids. The samples were incubated at 37°C for 30 min. After incubation, 2 μL of 6× Gel Loading Buffer (Thermo Fisher, Massachusetts, United States) was added to the samples, after which they were loaded onto a 1.5% agarose gel. Target dsRNA bands were visualized and photographed using a Gel Doc EZ Gel Documentation System (Bio-Rad, California, United States).

### Temporal and tissue expression profiles of *JHEH*, *JHDK*, and *MFE*

To evaluate the endogenous expression profiles of *JHEH*, *JHDK*, and *MFE* genes throughout the developmental stages of the CBW, three biological replicates were collected from each developmental stage. Each biological replicate was collected by pooling 15 first-instar larvae, 8 second-instar larvae, or 3 individuals from the other developmental stages (∼100 mg of tissue per replicate). Additionally, to compare the expression of *JHEH*, *JHDK*, and *MFE* between the carcass and gut tissues, tissue samples were dissected from third-instar larvae (five larvae per replicate, three replicates). All samples were frozen in liquid nitrogen and stored at −80°C for further RNA extraction, cDNA synthesis, and gene expression analysis.

### Insect bioassays *via* injection

Third-instar CBW larvae were dorsally injected with 2 µL of naked dsRNA, dsRNA + CS-TPP, or dsRNA + PEI. Three doses of ds*MFE*, ds*JHEH*, and ds*JHDK* were tested (0.05, 0.5, and 2 µg, respectively). Milli-Q ultrapure water (water, water + PEI, and water + CS-TPP), ds*GFP*, ds*GFP* + PEI, and ds*GFP* + CS-TPP were used as negative control treatments. The amount of naked ds*GFP* or ds*GFP* complexed with the nanoparticles injected into the insects was 2 µg. To evaluate gene silencing efficiency, three biological replicates were collected from each treatment 48 h after injection, immediately frozen in liquid nitrogen, and stored at −80°C until RNA extraction. Each biological replicate was formed by pooling three individuals. Once the optimal amount of dsRNA and the best type of nanoparticle for inducing silencing of the target genes in the CBW larvae were determined, further bioassays were performed to evaluate the effect of naked dsRNA and dsRNA-PEI on insect survival, phenotype, and gene silencing. In addition to the single dsRNA treatment, combinations of two dsRNAs were tested to evaluate whether the simultaneous silencing of multiple target genes increased insect mortality compared with the single-target strategy. The tested combinations of dsRNAs included ds*MFE*/*JHDK*, ds*MFE*/*JHEH*, and ds*JHDK*/*JHEH*. Third-instar larvae were injected with 500 ng of naked dsRNA, dsRNA-PEI, or a combination of the two types of dsRNAs (250 ng of each). The bioassays were repeated three times under the same conditions using 20 larvae per treatment (*N* = 60). For gene expression analyses, 20 larvae were injected with the tested treatments, and three biological replicates of three larvae were collected per treatment 48 h after injection. As a control group for each experiment, larvae were injected with the same amount of ds*GFP*. The injections were performed using a 700 Series Hamilton syringe (10 µL) (Hamilton Company, Nevada, United States) coupled with a 51-mm gauge 26S 4, 12° needle (Allcrom, São Paulo, Brazil).

### Insect bioassays *via* ingestion

The artificial diet was mixed with ds*GFP*, ds*JHEH*/*JHDK*, ds*GFP* + PEI, or ds*JHEH*/*JHDK* + PEI at two concentrations (100 and 1,000 ng of dsRNA per gram of diet). A total volume of 400 μL of the treated diet was applied to each microplate well (Thermo Fisher, Massachusetts, United States), and one newly hatched CBW larva was placed into the well. The larvae were transferred to new plates with the treated diet every 3 days and then fed an untreated diet from Day 15. Bioassays were repeated three times under the same conditions using 20 larvae per treatment (N = 60). For gene expression analyses, 20 larvae were fed on the tested treatments, and 4 biological replicates of three larvae each were collected per treatment 10 days after feeding. Phenotypic abnormalities and survival were recorded every day for 20 days. The representative images of the morphological alterations observed in the CBW insects upon dsRNA treatment were captured using a Leica DFC310 FX digital camera attached to a Leica MZ10 F stereoscope (Leica Microsystems, Wetzlar, Germany).

### RNA extraction and cDNA synthesis

Total RNA was isolated using TRIzol Reagent (Invitrogen, Massachusetts, United States) according to the manufacturer’s instructions. The integrity of the RNA samples was evaluated on a 1% (w/v) agarose gel through electrophoresis, and the RNA samples were quantified using a NanoDrop-2000 spectrophotometer (Thermo Fisher Scientific Inc., Massachusetts, United States). The RNA samples were treated with DNase I and RNase-Free (1 U/µL) (Invitrogen, Massachusetts, USA), and first-strand cDNA was synthesized from 2 µg of RNA using Oligo(dT)30 primers and M-MLV reverse transcriptase (Invitrogen, Massachusetts, United States) according to the manufacturer’s instructions.

### Gene expression analyses by RT-qPCR

Each RT-qPCR mixture contained 5 μL GoTaq qPCR Master Mix (Promega, Wisconsin, USA), 2 μL cDNA (diluted 20×), 2.6 μL nuclease-free water, and 0.4 μL of each forward and reverse primer (10 µm). *AgRPS26* and *AgRPS11* were used as reference genes. RT-qPCR was performed on a CFX96 Touch Real-Time PCR Detection System (Bio-Rad, California, United States) under the following conditions: initial denaturation at 95°C for 15 min, followed by 40 cycles of 95°C for 30 s, 60°C for 20 s, and 72°C for 30 s. Primer efficiency was calculated using the MINER software, and relative gene expression analysis was performed following the 2^−ΔΔCt^ method (Pfaffl, 2001) using the qbase^+^ software (Biogazelle, Gent, Belgium). The primers employed for the gene expression analysis are listed in Supplementary Table S1.

### Statistical analysis

Survival curves were generated using Kaplan‒Meier estimators, and the log-rank test was applied for pairwise comparisons between treatments. Gene expression differences among treatments were assessed by using one-way ANOVA with multiple comparisons (Tukey’s HSD). All statistical analyses were performed using the SPSS Statistics 27 software (IBM).

## Results

### Gene identification

The CBW full-length *MFE*, *JHDK*, and *JHEH* ORF’s (open reading frame) were 816, 1,371, and 615 bp, respectively. The analysis of the primary structure of each predicted protein revealed the presence of key signatures (Supplementary Figure S1). The conserved cytochrome P450 site located at 401–410 aa and a membrane anchor region at 5–22 aa were the main motifs found in the MFE sequence. Three GTP-binding motifs, DxN (40–43 aa), xxE (156–158 aa), and PGNFIFGx (194–201 aa), as well as three elongation factor hand motifs (calcium binding), were detected in the JHDK sequence. Last, the HGWP epoxide hydrolase domain (154–158 aa) and the catalytic triad (Asp225, Glu406, and His432) were identified in the JHEH sequence.

### Characterization of dsRNA–nanoparticle complexes

The mean particle size (d.nm) and surface charge, determined by DLS, varied widely between different proportions of CS:dsRNA and PEI:dsRNA (Table 1). For CS-TPP-dsRNA nanoparticles, increasing CS amounts led to smaller nanoparticles, while increasing PEI amounts in PEI-dsRNA complexes showed the opposite (Supplementary Figure S2). Regarding the distribution of particles, the smallest polydispersity coefficients (0.198 and 0.209) were detected for 5:1 and 6:1 (N:P) proportions of CS:dsRNA and PEI:dsRNA, respectively (Table 1). These proportions also presented the highest zeta potential (∼34 and ∼31 mV for CS nanoparticles and PEI nanoparticles, respectively), and no further increase was detected with higher amounts of any polymer (Supplementary Figure S3). Owing to their low PdI and considering that higher proportions increased particle size but not surface charge, while lower proportions presented lower charge, CS:dsRNA 5:1 and PEI:dsRNA 6:1 (N:P) were selected as optimal proportions and used for downstream analysis (including bioassays) in our study.

**TABLE 1:** Nanoparticle (50 ng/μL) characterization through dynamic light scattering analysis (DLS) using a Malvern Zetasizer Nano ZS instrument. Formulations CS:TPP:dsRNA (5:1:1) and PEI:dsRNA (N:P = 6:1) were used for downstream bioassays.

Sample	Size (d.nm)	SD±	Polydispersity (PdI)	SD±	Z potential (mV)	SD±
CS:TPP:dsRNA (1:1:1)	2,212	460.2	0.94	0.05	2.99	0.64
CS:TPP:dsRNA (5:1:1)	302	2.45	0.198	0.07	34.00	4.51
CS:TPP:dsRNA (10:1:1)	272	2.25	0.280	0.02	34.80	0.40
PEI:dsRNA (N:P= 1:1)	218	14.24	0.438	0.08	−3.27	1.67
PEI:dsRNA (N:P= 3:1)	247	1.37	0.283	0.03	6.31	0.77
PEI:dsRNA (N:P= 6:1)	219	4.70	0.209	0.02	31.00	3.22
PEI:dsRNA (N:P= 10:1)	421	11.45	0.369	0.02	30.20	0.47
PEI:dsRNA (N:P= 20:1)	437	24.44	0.581	0.08	31.70	5.05

### Stability of dsRNA in hemolymph and gut fluids of CBW larvae

**FIGURE 1:**
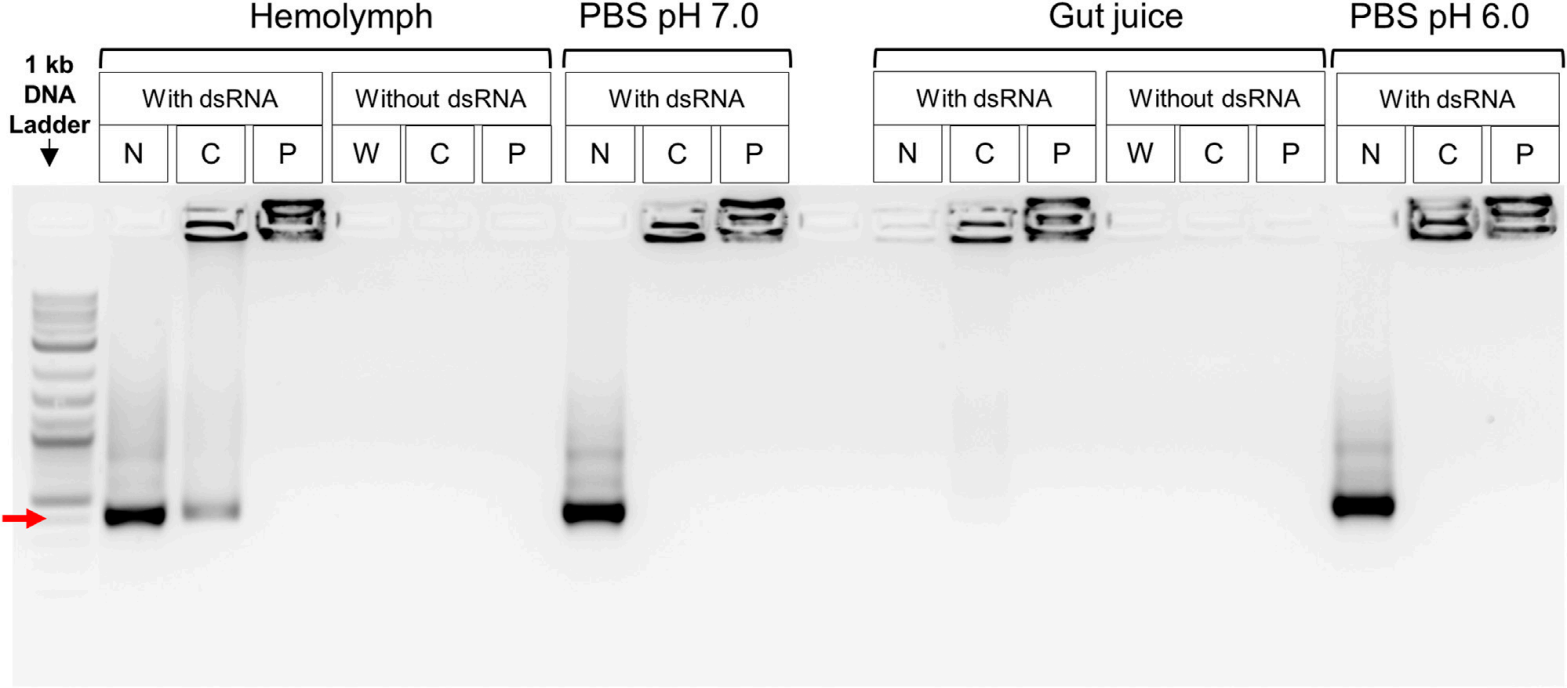
*Ex vivo* dsRNA degradation assay in CBW hemolymph and gut fluids. Red arrow indicates a 400 bp DNA fragment. N, naked dsRNA; C, chitosan–tripolyphosphate (CS-TPP) nanoparticle; P, polyethylenimine (PEI) nanoparticle; W, sample without dsRNA, CS-TPP, or PEI. Control treatments without dsRNA included insect fluid plus PEI or CS-TPP nanoparticles. Control treatments with dsRNA included PBS buffer plus PEI or CS-TPP nanoparticles complexed with dsRNA.

Degradation of the naked dsRNA was not observed after incubation with the CBW hemolymph, while a complete degradation of the dsRNA was induced by the CBW gut juice. The dsRNA complexed with CS-TPP or PEI nanoparticles was protected from the nuclease activity of the gut juice. Some fraction of the dsRNA was released from CS-TPP when incubated with the hemolymph and gut juice, but dsRNA-PEI complexes were stable in both body fluids. In addition, naked dsRNAs and nanoparticle-complexed dsRNAs were both stable in the PBS solution, and no degradation of the dsRNA was detected (Figure 1).

### Target gene expression profiles

**FIGURE 2:**
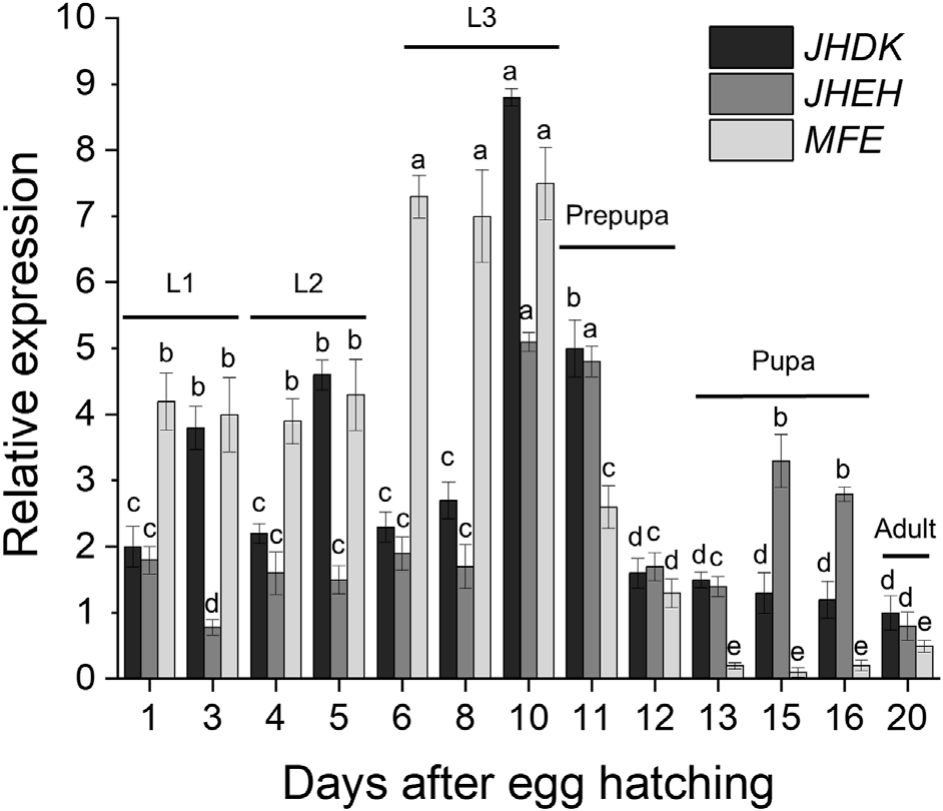
Expression profiles of target genes throughout the CBW developmental cycle. Relative expression is given as 2^−ΔΔCT^ (fold-change) values. Different letters indicate significant differences according to *p*-value <0.05 (one-way ANOVA followed by Tukey’s HSD). Comparisons are shown for individual genes and do not represent significant differences between the expression of different target genes. MFE, methyl farnesoate epoxidase; JHEH, juvenile hormone epoxide hydrolase I; JHDK, juvenile hormone diol kinase; L1–L3, larval instars.

The temporal and tissue expression profiles of *MFE*, *JHEH*, and *JHDK* were determined through RT-qPCR analysis. The expression of the *MFE*, *JHEH*, and *JHDK* genes was highly variable throughout the different developmental stages of the CBW (Figure 2). These genes were highly expressed primarily in larval and prepupal stages. Notably, the peaks in the expression of *JHEH* and *JHDK* were observed in the late third-instar larvae and early prepupal stages. The highest expression of *MFE* was also observed in the last larval stage; however, the *MFE* expression level remained similar throughout the third-instar larval stage, greatly decreased in the prepupa, and then gradually decreased from prepupa to pupa. *JHDK* and *MFE* expression was significantly higher in the carcass than in the gut (from 80% to 99% higher), whilst no significant difference in *JHEH* expression was observed between the carcass and gut (Supplementary Figure S4).

### RNAi response in the CBW upon injection of naked dsRNA and dsRNA nanoparticles

**FIGURE 3:**
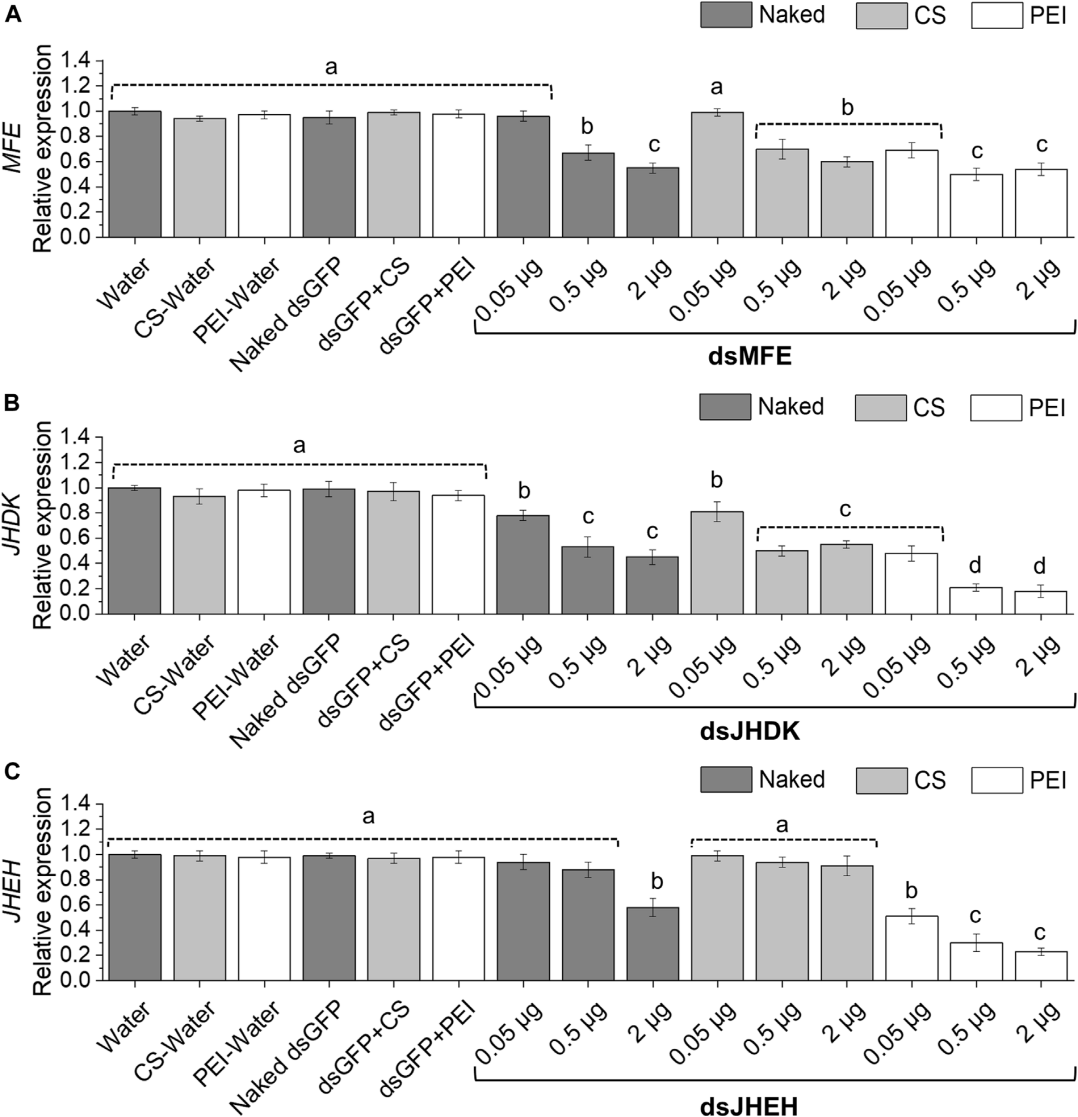
Expression of CBW target genes in response to different amounts of injected dsRNA. **(A)** *MFE* expression. **(B)** *JHDK* expression. **(C)** *JHEH* expression. Relative expression is given as 2^−ΔΔCT^ (fold-change) values. Gene expression in the water control was scaled to 1. Different letters indicate significant differences according to *p*-value <0.05 (one-way ANOVA followed by Tukey’s HSD). Data represent the mean ± SE (N: 12). Control larvae were injected with 2 µg of dsGFP. All injected larvae were 8 days old. Samples were collected 48 h after injection. MFE, methyl farnesoate epoxidase; JHEH, juvenile hormone epoxide hydrolase I; JHDK, juvenile hormone diol kinase; CS, chitosan–tripolyphosphate nanoparticles (CS-TPP); PEI, polyethylenimine nanoparticle; Naked, non-encapsulated dsRNA; GFP, green fluorescent protein.

Overall, our results showed that gene silencing levels were dose dependent. The amount of naked dsRNA required to induce significant gene silencing was variable, depending on the target gene. All dsRNA doses tested led to a significant reduction in *JHDK* expression, while 0.5 or 2 µg of ds*MFE* repressed *MFE* expression (Figures 3A, B). However, only the dose of 2 µg of ds*JHEH* silenced the target gene (Figure 3C). Surprisingly, CS-TPP-dsRNA complexes induced the same level of gene silencing as the naked dsRNAs and, in some cases, impaired or reduced the level of gene silencing. On the other hand, PEI nanoparticles complexed with dsRNA at all tested doses improved *JHDK* and *JHEH* gene silencing compared with naked dsRNA. Although ds*MFE* + PEI did not lead to significant enhancement in the level of gene silencing when applied at 2 µg, when compared with naked dsRNA, ds*MFE* + PEI at low and moderate doses (0.05 and 0.5 µg, respectively) induced significantly greater gene silencing than the naked dsRNA at the same doses. Among the treatments involving PEI, the highest levels of *MFE*, *JHDK*, and *JHEH* silencing were achieved with moderate (0.5 µg) and high (2 µg) doses of dsRNA-PEI (Figure 3). Finally, persistent silencing of the *MFE*, *JHDK*, and *JHEH* genes was observed in the larvae injected with the respective naked dsRNAs or dsRNA-PEI at 0.5 µg. Significant differences in the expression of the target genes were detected between the treated larvae and control group up to 96 h after injection (Supplementary Figure S5).

As the RNAi response of the CBW was enhanced by PEI nanoparticles but not by CS-TPP, our subsequent experiments were performed only with PEI nanoparticles. In addition, as the dsRNA dose of 0.5 µg induced the same level of gene silencing as a 2 µg dose when the dsRNA + PEI complex was injected into the larvae, we selected the 0.5 µg dose for subsequent bioassays.

**FIGURE 4:**
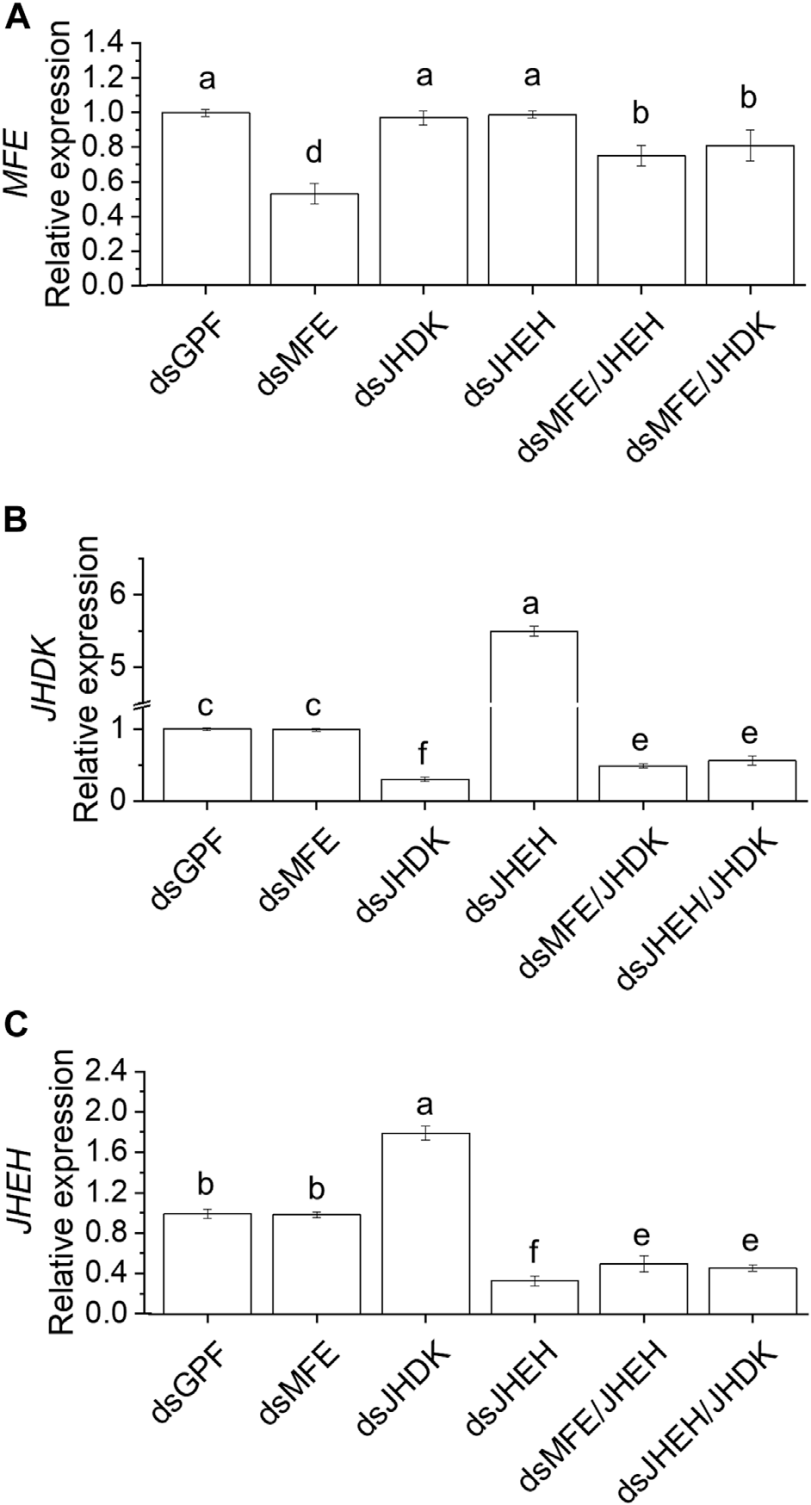
Comparison of RNAi-mediated silencing efficiency induced by a single dsRNA or combination of dsRNAs. **(A)** *MFE* expression. **(B)** *JHDK* expression. **(C)** *JHEH* expression. All dsRNAs used were complexed with PEI. Relative expression is given as 2^−ΔΔCT^ (fold-change) values. The gene expression in the ds*GFP* control was scaled to 1. Different letters indicate significant differences according to *p*-value <0.05 (one-way ANOVA followed by Tukey’s HSD). Data represent the mean ± SE (N: 12). Samples were collected 48 h after injection. Eight-day-old larvae were injected with 500 ng of dsRNA. MFE, methyl farnesoate epoxidase; JHEH, juvenile hormone epoxide hydrolase I; JHDK, juvenile hormone diol kinase; GFP, green fluorescent protein; PEI, polyethylenimine nanoparticle.

We further investigated the effects of delivering multiple dsRNAs complexed with PEI nanoparticles on target gene expression (Figure 4). Gene silencing efficiency was significantly higher when single dsRNAs were injected into the insects than when a combination of dsRNAs were injected. The silencing of *JHEH* or *JHDK* did not induce transcriptional changes in *MFE* (Figure 4A). However, *JHDK* silencing induced upregulation of *JHEH* and *vice versa* (Figures 4B, C). Furthermore, simultaneous silencing of *JHDK* and *JHEH* prevented the upregulation of either gene.

**FIGURE 5:**
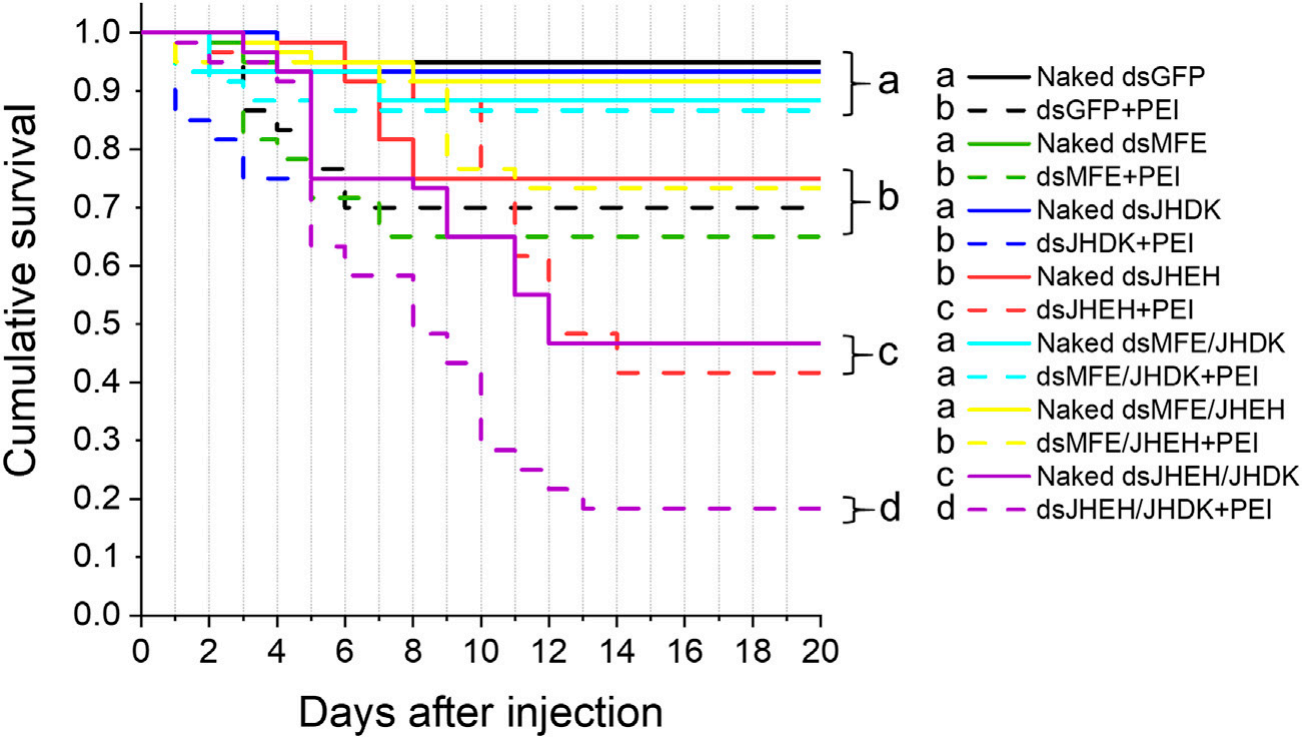
Survival curves of larvae injected with different formulations of dsRNA. Eight-day-old larvae were injected with 500 ng of dsRNA. Survival curves were generated using the Kaplan‒Meier estimator. Different letters indicate significant survival differences between groups (log-rank test, p-value <0.05; N: 60). MFE, methyl farnesoate epoxidase; JHEH, juvenile hormone epoxide hydrolase I; JHDK, juvenile hormone diol kinase; GFP, green fluorescent protein; PEI, polyethylenimine nanoparticle; Naked, non-encapsulated dsRNA.

**FIGURE 6:**
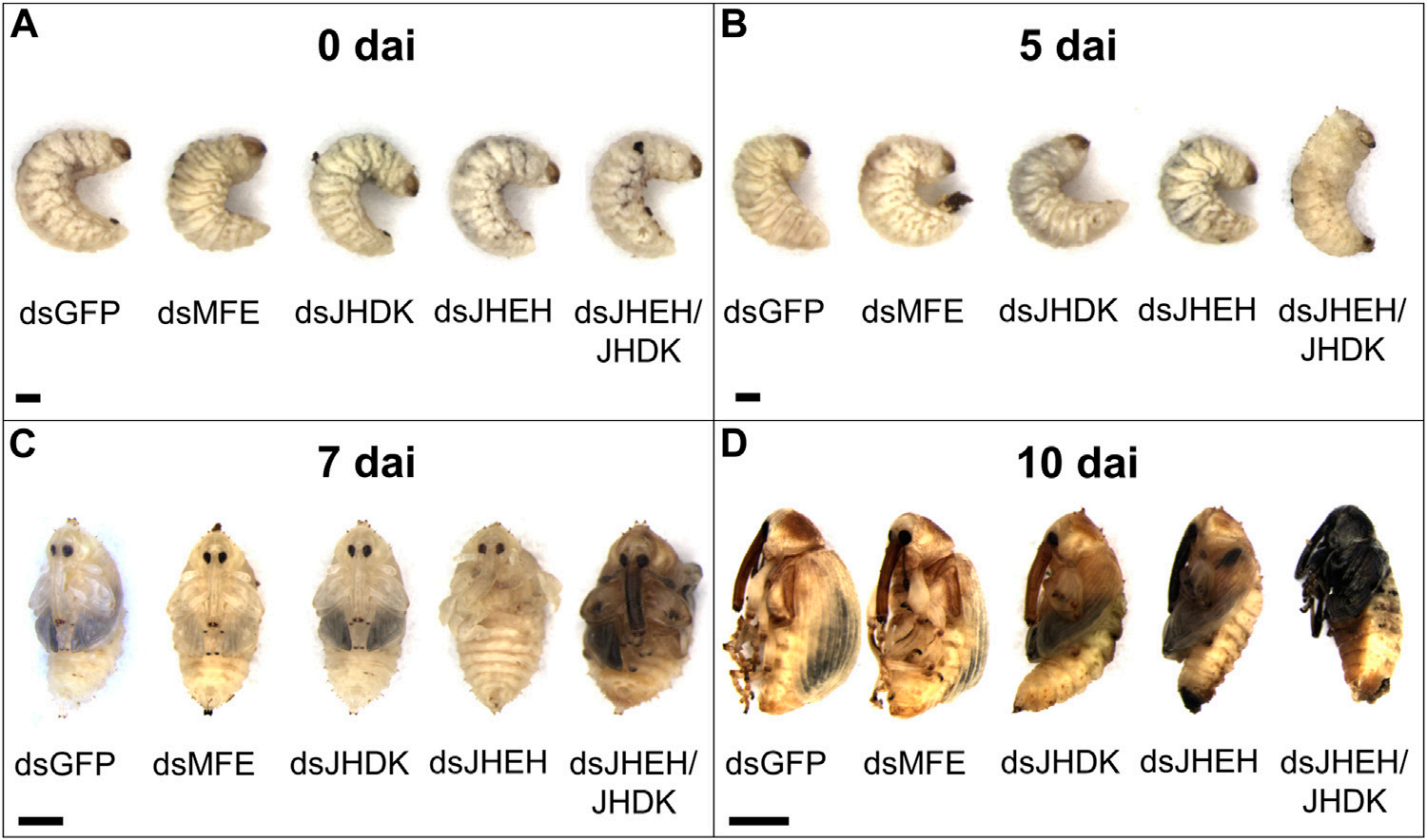
Phenotypic alterations associated with the silencing of target genes in CBW larvae. **(A)** Early third-instar larvae. **(B)** Prepupa. **(C)** Early pupa. **(D)** Early adult. MFE, methyl farnesoate epoxidase; JHEH, juvenile hormone epoxide hydrolase I; JHDK, juvenile hormone diol kinase; GFP, green fluorescent protein; dai, days after dsRNA injection. Scale bar, 1 mm.

Regarding the effect of naked dsRNAs and PEI nanoparticles on CBW development and survival, we found that the ds*JHEH*/*JHDK* + PEI (18%), ds*JHEH* + PEI (42%), ds*JHEH*/*JHDK* (47%), and ds*JHEH* (75%) treatments induced significantly lower survival rates than the respective ds*GFP* + PEI (70%) and ds*GFP* (95%) controls (Figure 5). These results demonstrated that PEI nanoparticles and the combination of certain dsRNAs reduced CBW survival and affected its development (Figure 6). However, this result was not consistent across all target genes tested in this study. In fact, only the injection of third-instar larvae with ds*JHEH*/*JHDK*, naked or complexed with PEI, resulted in malformed adults. Malformed individuals showed an incomplete transition to the adult stage. When compared with normal adults, malformed insects had smaller elytra, which were completely tanned and hardened but positioned on the ventral side rather than on the dorsal side. This phenotype was not observed in any of the remaining treatments. However, most surviving individuals treated with dsRNA targeting *JHEH* (65%) or *JHDK* (54%) presented delayed adult development (Figure 6D).

### RNAi response in the CBW upon feeding with naked dsRNA or dsRNA-PEI

**FIGURE 7:**
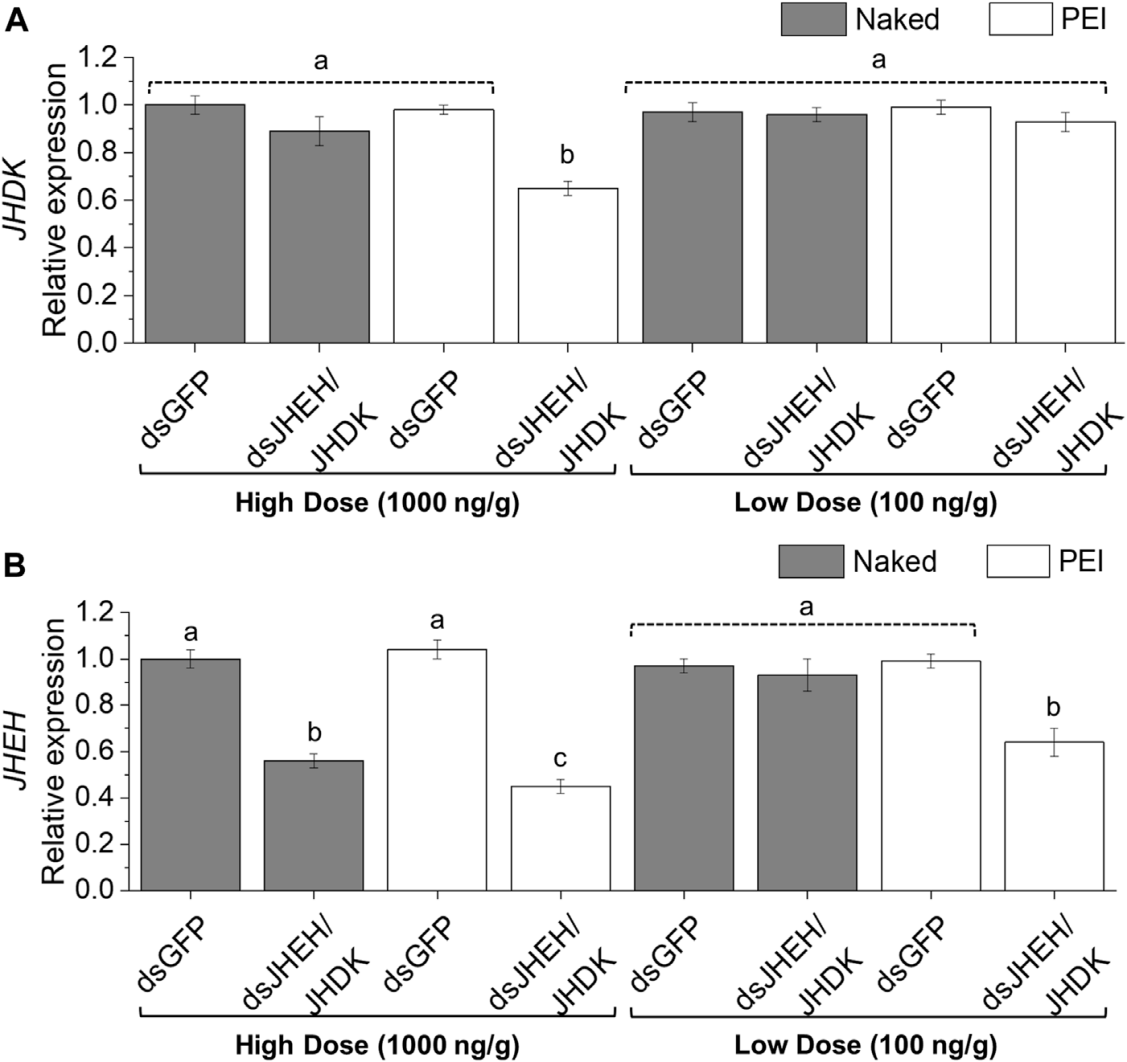
Expression of CBW target genes in response to combined dsRNA formulations delivered by feeding. **(A)** *JHDK* expression. **(B)** *JHEH* expression. Relative expression is given as 2^−ΔΔCT^ (fold-change) values. The gene expression in the ds*GFP* control was scaled to 1. Different letters indicate significant differences according to *p*-value <0.05 (one-way ANOVA followed by Tukey’s HSD). Data represent the mean ± SE (N: 12). Dosage is given as nanograms of dsRNA per milligram of diet. Samples were collected 10 days after the first diet delivery to neonate larvae. JHEH, juvenile hormone epoxide hydrolase I; JHDK, juvenile hormone diol kinase; GFP, green fluorescent protein; PEI, polyethylenimine nanoparticle; Naked, non-encapsulated dsRNA.

We further investigated the possibility of using PEI nanoparticles as carriers to deliver dsRNA to the CBW through feeding. The combination of ds*JHEH* and ds*JHDK*, which induced the lowest survival rate when delivered by injection, was chosen for testing in feeding bioassays. The expression of *JHDK* was reduced significantly (55%) in larvae that were fed ds*JHEH/JHDK* + PEI at 1,000 ng/g compared with the ds*GFP* + PEI control. However, neither ds*JHEH*/*JHDK* nor ds*JHEH*/*JHDK* + PEI at 100 ng/g or ds*JHEH*/*JHDK* at 1,000 ng/g induced *JHDK* silencing (Figure 7A). On the other hand, CBW larvae that were fed ds*JHEH*/*JHDK* and ds*JHEH*/*JHDK* + PEI at 1,000 ng/g and ds*JHEH*/*JHDK* + PEI at 100 ng/g showed up to 56% *JHEH* silencing compared with the controls (Figure 7B).

**FIGURE 8:**
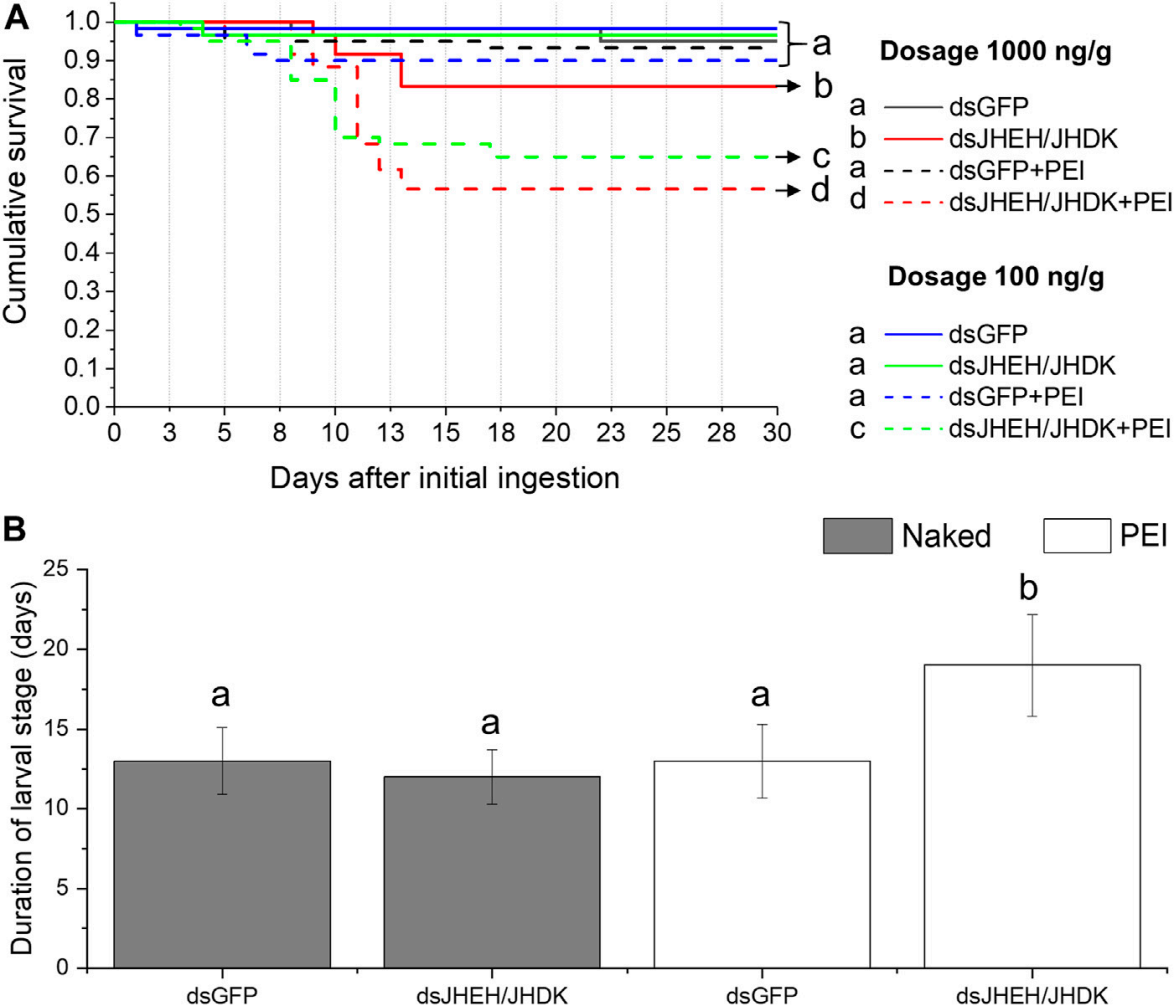
Effect of combined dsRNA formulations on CBW survival and development. **(A)** Cumulative survival of individuals treated with two different concentrations of dsRNA delivered by feeding. Survival curves were generated using the Kaplan‒Meier estimator. Different letters indicate significant survival differences between groups (log-rank test, *p*-value <0.05; N: 60). **(B)** Average duration of the larval stage in individuals treated with 1,000 ng/g dsRNA formulations. Different letters indicate significant differences according to p-value <0.05 (one-way ANOVA followed by Tukey’s HSD). Data represent the mean ± SE (N: 30). JHEH, juvenile hormone epoxide hydrolase I; JHDK, juvenile hormone diol kinase; GFP, green fluorescent protein; Naked: non-encapsulated dsRNA; PEI, polyethylenimine nanoparticle.

Both low- and high-dose ds*JHEH*/*JHDK* + PEI significantly reduced the survival rates (56%–65%) of CBW compared with the controls (90%–93%). Furthermore, naked ds*JHEH*/*JHDK* at 1,000 ng/g resulted in slightly lower survival than that observed in the controls (Figure 8A). Surprisingly, larvae that were fed ds*GFP* + PEI did not exhibit significant mortality, as previously observed in the injection bioassays. In addition, we found that the larval stage duration of surviving insects that were fed ds*JHEH*/*JHDK* + PEI at 1,000 ng/g was significantly longer than that of the control group (Figure 8B). With respect to developmental alterations, the abnormal phenotypes observed in insects that were fed ds*JHEH*/*JHDK* + PEI were the same as those that were associated with simultaneous silencing of *JHEH* and *JHDK* induced by dsRNA injection (Figure 6).

## Discussion

Juvenile hormone regulates insect molt and metamorphosis along with ecdysteroids. In the presence of JH, ecdysteroids trigger larval–larval molt, while in the absence of JH, ecdysteroids direct larval–pupal–adult metamorphosis (Liu et al., 2018). Thus, any changes in the hemolymph levels of enzymes that participate in JH biosynthesis or degradation should interfere with JH levels and, consequently, with the biological processes controlled by this hormone. In this study**,** we demonstrated that simultaneous silencing of *JHEH* and *JHDK*, two genes encoding enzymes involved with JH degradation, compromised CBW molting, resulting in adult insects with an abnormal phenotype that eventually died.

In agreement with our results, the injection of *Apolygus lucorum* third-instar nymphs with dsRNA targeting the *JHEH* gene significantly decreased the survival rate and blocked the molting process (Tusun et al., 2017). Furthermore, *JHEH* silencing in *Plutella xylostella* larvae induced significant mortality, exceeding 66% and demonstrating the role of this gene in insect development (Chaitanya et al., 2017). Unexpectedly, in our study, *JHDK* silencing in CBW larvae did not cause significant mortality or any visible abnormal phenotype. By contrast, *JHEH* knockdown induced insect mortality without causing clear morphological abnormalities. However, the simultaneous knockdown of *JHEH* and *JHDK* genes greatly decreased the survival of CBW and resulted in severe malformations in the adults. Interestingly, we observed that the silencing of *JHEH* triggered the overexpression of *JHDK* and *vice versa*, suggesting a compensatory mechanism at the transcriptional level for the regulation of the same metabolic pathway. Similarly, a previous study showed that the suppression of *JHDK* in *Heortia vitessoides* upregulated *JHEH* expression (Lyu et al., 2019). However, this study reported that the RNAi-mediated silencing of *JHDK* resulted in increased larval mortality and defective phenotypes, in contrast to our data. In addition, it is important to note that continuous dsRNA feeding resulted in delayed development at the larval stage of individuals in which *JHEH* and *JHDK* were simultaneously silenced. In a previous study, the same outcome was observed in *Bombyx mori* larvae after the knockout of another JH-degrading enzyme, JH esterase (Zhang et al., 2017).

Two main JH degradation pathways have been described. In one potential route, JH is converted to JH acid by JH esterase (JHE), followed by conversion to JH acid diol by JHEH. In an alternative route, JH is converted to JH diol by JHEH, followed by conversion to JH acid diol or JH diol phosphate by JHE or JHDK activity, respectively (Maxwell et al., 2002a, 2002b). While JHEH is involved in both pathways, JHDK may participate in only one. We hypothesize that the silencing of *JHDK* alone failed to induce mortality or abnormal phenotypes in the CBW because even after the silencing of *JHDK*, JH degradation might occur through alternative routes to maintain normal levels during insect development. Based on the two-degradation route model for JH (Morgan, 2010), our data suggest that JHEH and JHDK play a role in the CBW molting process through different degradation routes that can compensate each other. However, quantification of JH titers in CBW’s hemolymph after *JHEH* and *JHDK* knockdown or knockout is necessary to elucidate their participation in the JH degradation route. To date, the detection of JH in the CBW has been performed by radiochemical assays (Taub-Montemayor et al., 1997; Taub-Montemayor et al., 2005). This approach has been substituted by modern techniques, such as high-performance liquid chromatography–mass spectrometry (HPLC-MS) and gas chromatography–mass spectrometry (GC-MS), due to their higher safety and resolution capacity. Nevertheless, most of these studies have been performed on Lepidoptera, Diptera, and Hemiptera, while for Coleoptera, there is little information (Westerlund and Hoffmann, 2004; Kai et al., 2018; Ramirez et al., 2020). Thus, the optimization of modern methods to detect and quantify JH in coleopterans’ hemolymph is a key step to performing functional validation of genes involved with JH metabolism.

In addition, it is not clear whether the JHEH isoform that acts on JH acid is the same as the one that binds to JH. We have raised two hypothetical scenarios to explain our results: in the first, our dsJHEH molecule can affect both pathways either by knocking down a single gene involved in both routes or by knocking down two different isoforms. In the second, our dsJHEH molecule affected only the gene expression involved in JH acid degradation, thus blocking only the JHE-dependent pathway. Whatever the case, further studies are required to fully understand the biological roles of these enzymes and their compensatory and synergistic actions in the JH degradation pathway in the CBW.

Molting-related deformities and significant mortality were not observed in the CBWs in which the *MFE* gene was silenced. Nouzova et al. (2021) demonstrated that MF epoxidase (EPOX) (also called CYP15C1)–null mutant *A. aegypti* larvae successfully complete metamorphosis, reach adulthood, and reproduce, suggesting that *epox* is not an essential gene in mosquitos. However, EPOX-deficient mosquitoes suffer a significant reproductive fitness cost. A recent study showed that the RNAi-mediated reduction of *CYP15C1* expression in *C. suppressalis* results in increased larval mortality compared with the control groups. In addition, incomplete ecdysis, melanization in the head and thorax, and delayed development were observed (Sun et al., 2020). Our results indicated that *MFE* suppression does not compromise CBW development, as CBW metamorphosis and survival were not affected. Nevertheless, CRISPR/Cas9-mediated knockout of *MFE* could provide stronger evidence that epoxidated JH is not essential for larval–pupal–adult metamorphosis in the CBW, as it is possible that RNAi treatment does not reduce *MFE* expression sufficiently to induce visible abnormal or lethal phenotypes.

Regarding the use of nanoparticles, we compared two polymers, CS (using TPP as cross-linker) and PEI, as nanocarriers for dsRNA delivery. Although both polymers produced nanoparticles with similar characteristics (surface charge, size, and polydispersity), they were stable in the fluids of the CBW (Figure 1). Complexes of CS-TPP-dsRNA did not induce higher gene knockdown than naked dsRNA, while PEI-dsRNA complexes strongly silenced target genes. Although the potential of CS to stabilize dsRNA molecules has been demonstrated in a number of studies, most of them were performed on mosquitoes (Diptera) and lepidopteran larvae (Das et al., 2015; Kumar et al., 2016; Dhandapani et al., 2019; Gurusamy et al., 2020; Kolge et al., 2021). To date, there is no evidence that CS improves gene silencing in coleopteran pests. In addition, little is known about how nanoparticles are imported by cells of different insect species and how dsRNA is released once inside cells. We emphasized the importance of testing different polymers as nanocarriers and noted that nanoparticle stability should not be the only parameter for efficiency. In order to take a step further in the development of a nanoparticle-mediated delivery of siRNA/dsRNA, other factors, such as cell import, extracellular transport, and intracellular release, must be evaluated in future studies.

We observed that the delivery of dsRNA complexed with PEI nanoparticles resulted in improved gene silencing and lower survival rates than that observed with naked dsRNA. In comparison with the ds*JHEH* and ds*JHEH*/*JHDK* treatments delivered by injection, ds*JHEH* + PEI and ds*JHEH/JHDK* + PEI decreased insect survival by 33% and 29%, respectively. Similarly, survival decreased by 30% in the insects that were fed ds*JHEH/JHDK* + PEI compared with insects fed with the same combination of naked dsRNAs. Our data demonstrate the possibility of inducing a stronger RNAi response in the CBW using dsRNA complexed with PEI as a nanocarrier, which proved to be a viable tool for functionally characterizing CBW genes. The control ds*GFP* + PEI treatment caused significant mortality compared with naked ds*GFP* when injected into the CBW larvae, suggesting a non-specific toxic effect of this formulation in the CBW. This undesirable mortality was not observed in the feeding bioassays, indicating a possible detoxification mechanism in the gut cells of the CBW.

We found that PEI nanoparticles protect dsRNA from degradation caused by the nucleases present in the gut fluid. Thus, the increased RNAi response observed in CBW treated with dsRNA-PEI in feeding bioassays appears to be partially associated with the protection of dsRNA by PEI. Given that we did not detect degradation of naked dsRNA by the CBW hemolymph, we speculate that the stronger RNAi effects observed in the insects injected with dsRNA-PEI might be related to increased dsRNA cellular uptake. Whether PEI nanoparticles improve dsRNA cellular uptake efficiency remains to be investigated. Several dsRNA delivery systems have been proposed to increase RNAi efficiency in insects (Avila et al., 2018; Christiaens et al., 2018; Jain et al., 2022). Our data showed that an enhanced RNAi effect was achieved in the CBW when PEI nanoparticles were used as the carriers of dsRNA. Therefore, future loss-of-function studies might employ these nanoparticles to facilitate the analysis of the biological roles of other genes.

In summary, our study indicates that PEI nanoparticles could be used as an alternative approach to deliver dsRNAs to the CBW *via* either injection or feeding for gene functional analyses. Furthermore, we have demonstrated that simultaneous silencing of *JHEH* and *JHDK* causes significant mortality and ecdysis-related deformities, indicating that these genes could be employed in the development of RNAi-based methods for controlling the CBW.

## Data Availability

The original contributions presented in the study are included in the article/Supplementary Material; further inquiries can be directed to the corresponding author.
