# Supplementary material for: Simultaneous silencing of juvenile hormone metabolism genes through RNAi interrupts metamorphosis in the cotton boll weevil
Source: Front Mol Biosci. 2023 Mar 6;10:1073721. doi: 10.3389/fmolb.2023.1073721 (PMC10025338; doi:10.3389/fmolb.2023.1073721)
Supplement: Supplementary file 1 [file DataSheet1.PDF]

Supplementary Material

Supplementary Data

A. Juvenile hormone diol kinase gene from *Anthonomus grandis*

Source: transcriptome assembly from *A. grandis* larvae

>Contig21071_*Agjhdk*-like

atggaccacaagtggaccaacgcgatcaaagtgggcctgattgtgaaccgtttggccaacatgcccatctccgagtttcgcaagaagaagctcctc
tacgtcttcaacgtgtttttcgatgttaatcagagcggtacgatagatcgcaaggacttcgagttggccatagagaaaatttgcaccctaaggggctgg
ccgcaggggagcgaggagtacaagagaacgtacgacagcatgatccagatatgggagggactgagacagaaggccgactccaataaggacg
gacaagtgagcgtggaggagtgggccaccatgtgggacgactactccaagaacccggaaaacgccctggagtggcagaaccagtaccggaag
ttcatgttcgacctggaggacgcgagcggggacggcggtatcgatatcgacgagttcaccgcggtctgttcctgttacggtttggaaactggagagt
gccgggaggctttcgagaaaatggccgggggcaggaacctggtcgactatcaacagttccaggcgctctggcagcagtttttcatgtccgaggac
cagtccgaacccggaaacttcatcttcggcaagaccaagttttag

B. Juvenile hormone epoxide hydrolase I gene from *Anthonomus grandis*

Source: transcriptome assembly from *A. grandis* larvae

>Contig2479_*Agjheh*-I

atggctctactgaaatttcatataattgctatcgtcagtgttattataggaattttattgtataaactaactagtccaggtcctgtgccaacatttgataagaa
ccagtactggggacccggagaacccgctgcaaatcctgataatgcagtgcgaaagttcaccattgacattcccaccaaagaacaggaagatcttaa
atggagactggagcatgtaaggcccttggcacctcccttagaaggtgttcaacagcagtacggaatgaacacgaacctcctgaaagagatattgga
attctggagaacccagtataactggactgagaggcaacagtatctcaaccagtatccacaatttaaaacgaacatccaagggctgaatatccattttct
ccatgtgaaacccgagaatccaggcaacaaaaaggtgcttccgttattgattgtccacggttggcctggatcggtcagggagttttatgacgtcataa
aaatcttgactaaagtcagcgatgacagggatttcgtgtttgaggtggtggctcctcatattcctggattcgggttttctcagggggctgccaaacctg
gattgggttccaatcagatcgccgtcataatggcgaaccttatggaaaggttggggtataaggaattttattgtcaaggtggcgatttcggcgcaatca
tcttacaagccctctcggtaatctatccggaaaaagtcctggggtaccacaccaacatggcgtacgtcagcaccccttgggcgtacataaaaacctt
cttggggcaactttaccccacgtgggtggtgaaaccggaacaccataaccgggtttatccgttatgggaaaaattccaacacaggatcttggaaacc
ggatatttacatctgcaagccaccaagccggacactttaggtgtggctttaaccgattcacctgccggtttggccgcttatattctggaaaagttcacca
cgggcaccaacaggaatttccgatatagagaggacggaggattgctattaaaatacacttacacagaccttgtggataacgttatgttgtactggtgg
acctccagcgccaccacctcagttagactgtatgcagagagtttcagtaacagacatatggatgagaacgtccttaagactcctttgagagtacccac
agccatagcgaggttcacctatgatttctacgtggccgatggacttttgggggaaatttaccctaataacttgcagctaagtgacctagagggtggtca
ttttgctgccttcgaggtgccgggagttttagccaaagacgtttttctgccgtgcagaaatttgaagaataccaaaagaataggaaataa

C. Methyl farnesoate hydrolase gene from *Anthonomus grandis*

Source: transcriptome assembly from *A. grandis* larvae

>Contig31375_*Agmfe*-like

Atgcacagggcctttgacgtgtccgtcctaaacgccttgtgggccatgctggcaggggtacgttttgacgtcaacgacgcccgcctaaccgaactc
ctccatatcctccacgactgtttccggatagtggacatatccggaggtctgatcaaccaaatgcccatcttgaggttcgtggctccggacgccactgg
atacaaccagttcctggacgtgctcaataggatgtggacctttttgcaggaaacaattgaggagcaccgaaggaccatcccgccccaccctaggga
cctcattgacgcctttttgaagtccatggaggtcaaggtggatgactcgtttacagatgatcaactggtgtcactttgcttggatctgtttatggctgggg
cggagaccactagcaacatcttaggtttcaccagcatgtatatggtgctatatccggaggtgcagaggaaggtccagaaggagctcgacgatgtgg
tggggagggaccgctggcccaccattaaggacaaacccaatctgacttatacgcaagcagtactcctggaaatcctcaggagaaccgacctggc
gcccatgagcatcgcgcacagggccaccaaagatactcaactaatgggatatgacatcgctgaggacaccatcattttgactaatttacacagtgtc
cacatgaacgaggaggtctatgaaaaccccgaggagttcctgccagagaggttcatcggagataacgggaggatagtggtgaacgagaagacct
tcatccccttcgggttcgggaagaggcgctgcctgggggaggtg

Supplementary Data S1. Sequences and accession numbers of target genes used for the design of dsRNA. Sequence highlighted in red was used as template for dsRNA synthesis.

Supplementary Tables and Figures

Supplementary Table S1. Primer list for RT-qPCR reactions.

Species	Gene	Primers (5'--3')		Fragment length (bp)
		Forward	Reverse	
A. grandis	*RPS11*	ACCCACGGTTTTCCTTAACC	GACGACTCCGGTGAGGATAC	178
A. grandis	*RPS26*	TCCCAAAAGACAAGGCAATC	CCTTCTTGCTCCTGTTACGC	183
A. grandis	*MFE*	ATATGACATCGCTGAGGACAC	GGTCTTCTCGTTCACCACTATC	142
A. grandis	*JHDK*	ACGGTTTGGAAACTGGAGAG	TGGTCTTGCCGAAGATGAAG	153
A. grandis	*JHEH*	TACATCTGCAAGCCACCAAG	TAGCAATCCTCCGTCCTCTC	145

Supplementary Table S2. Composition of artificial diet used for rearing *Anthonomus grandis* in laboratory conditions.

COMPONENT	AMOUNT (gr)
Brewer's yeast	60
Soybean protein	100
Sugar	60
Wheat germ	60
Vitamins*	10 mL
Nipagin	2
Salt (Mineral)	10 g
Sorbic acid	2.4
Agar	40
Pharmamedia	40
Ascorbic acid	20
Distilled water	1.5 L

*Niacin; calcium pantotenate; riboflavin; tiamin; piredoxin; folic acid; biotin; cyanocobalamine; vitamin B12 e distilled water.

A

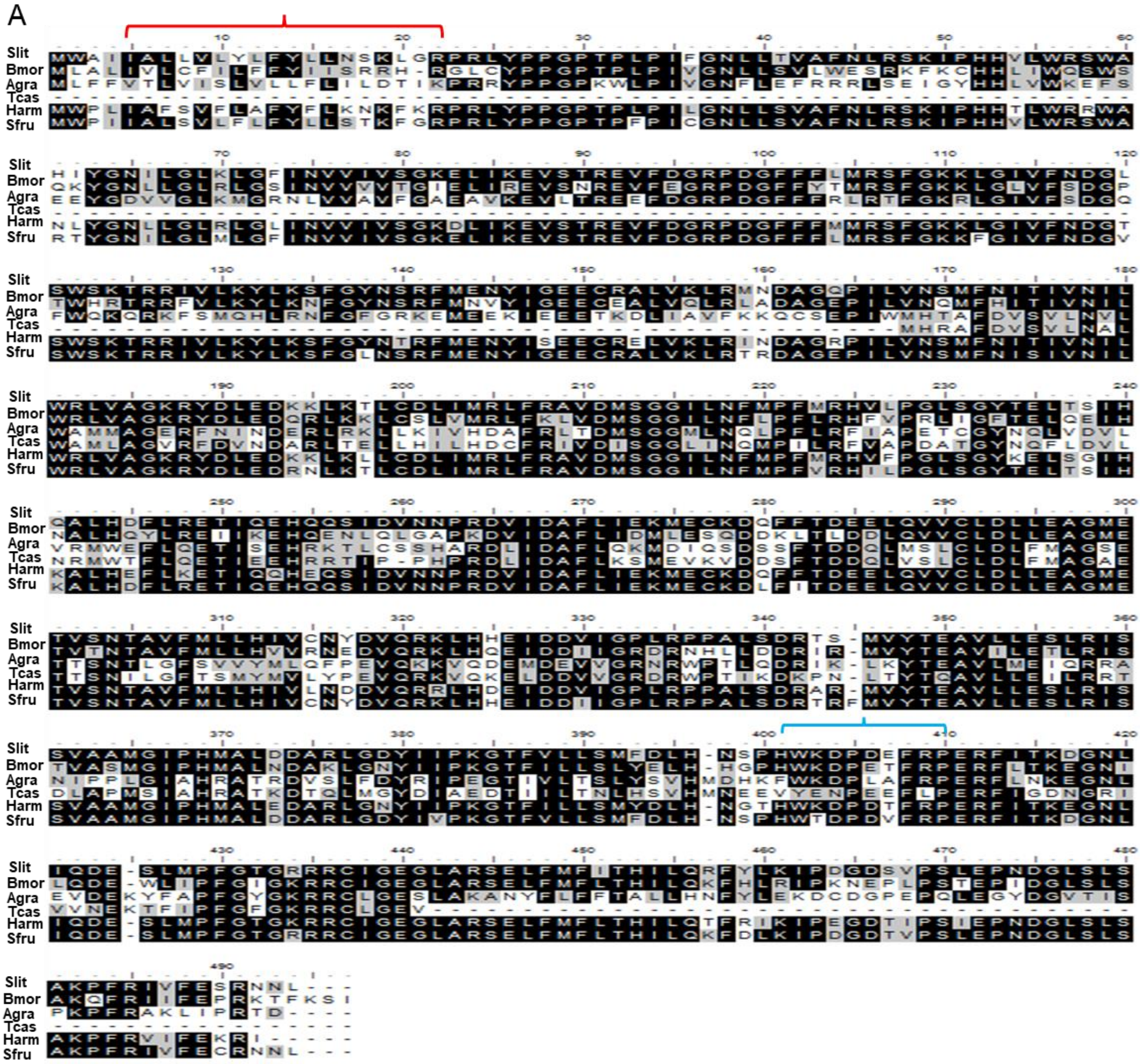

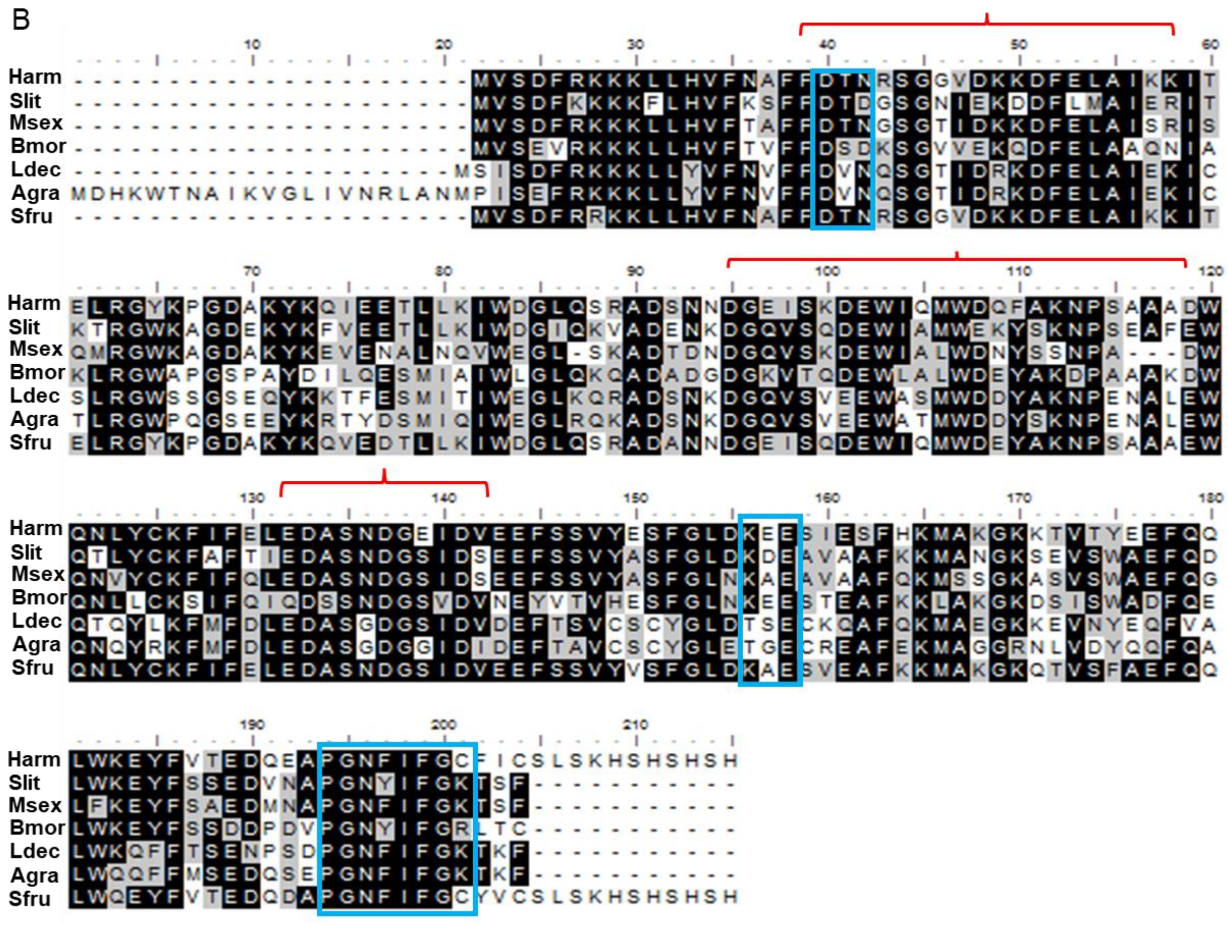
B
Harm
Slit
Msex
Bmor
Ldec
Agra
Sfru

C

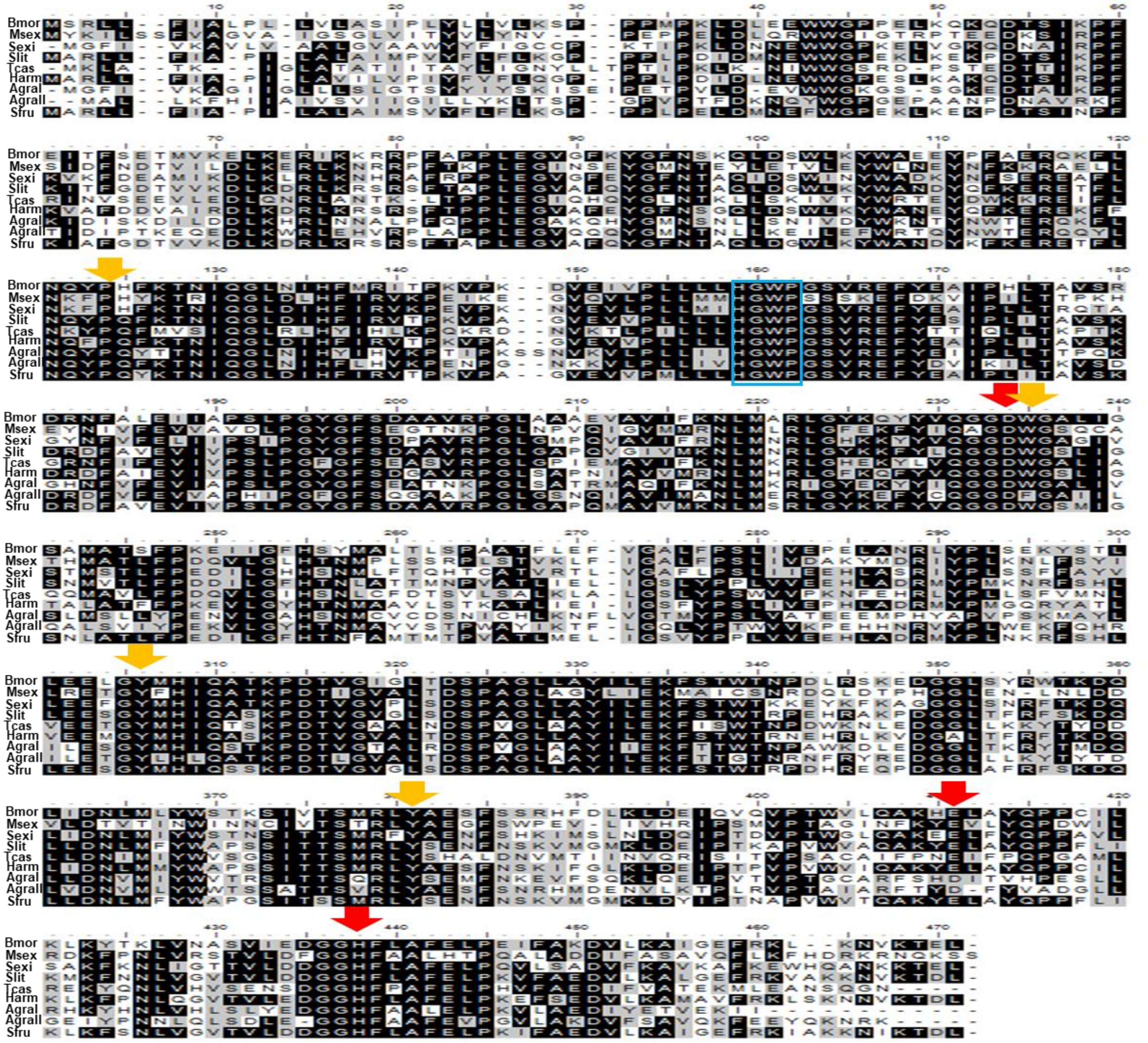

Supplementary Figure S1. Primary structure characterization and key signatures of the predicted protein sequences from target genes of *A. grandis*. **A)** *AgMFE*. Slit: *Spodoptera littoralis* (XP_022830043), Bmor: *Bombyx mori* (NP_001140197), Agra: AgMFE, Tcas: *Tribolium castaneum* (EFA01264.1), Harm: *Helicoverpa armigera* (XM0213257831), Sfru: *Spodoptera frugiperda* (GSSPFG00020555001.2). Blue brace indicates the cytochrome P450 conserved site and red brace the membrane anchor region **B)** *AgJHDK*. Harm: *Helicoverpa armigera* (XM0213353041), Slit: *Spodoptera littoralis* (AKN89772.1), Msex: *Manduca sexta* (CAD23378.1), Bmor: *Bombyx mori* (NP001037080.1), Ldec: *Leptinotarsa decemlineata* (AKF11872.1), Agra: *Anthonomus grandis* JHDK, Sfru: *Spodoptera frugiperda* (GSSPFG0030383001). Red braces indicate GTP binding motifs and blue boxes elongation factor -hand motifs (calcium-binding). **C)** *AgJHEH*. Bmor: *Bombyx mori* (AAQ97024.1), Msex: *Maduca sexta* (AAC47018.1), Sexi: *Spodoptera exigua* (ABD85119.1), Slit: *Spodoptera littoralis* (XP022837563), Tcas: *Tribolium castaneum* (NP001161904), Harm: *Helicoverpa armigera* (XM0213437261), AgraI: *Anthonomus grandis* JHEH I, AgraII: *Anthonomus grandis* JHEH II, Sfru: *Spodoptera frugiperda* (GSSPFG00024449001). Blue box indicates hydrolase domain and red arrows the catalytic triad.

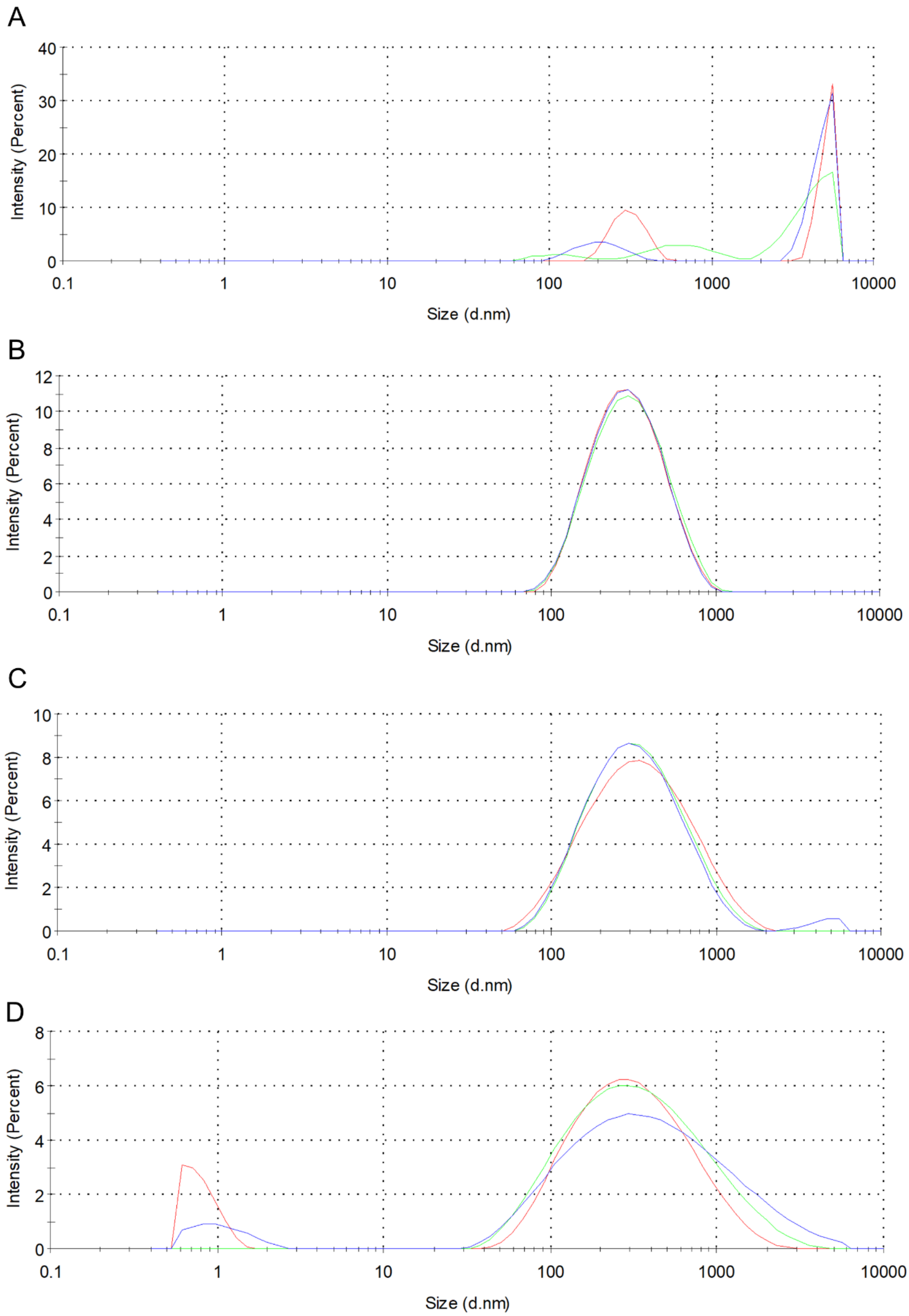
A
Intensity (Percent)
40
30
20
10
0
0.1
1
10
100
1000
10000
Size (d.nm)
B
Intensity (Percent)
12
10
8
6
4
2
0
0.1
1
10
100
1000
10000
Size (d.nm)
C
Intensity (Percent)
10
8
6
4
2
0
0.1
1
10
100
1000
10000
Size (d.nm)
D
Intensity (Percent)
8
6
4
2
0
0.1
1
10
100
1000
10000
Size (d.nm)

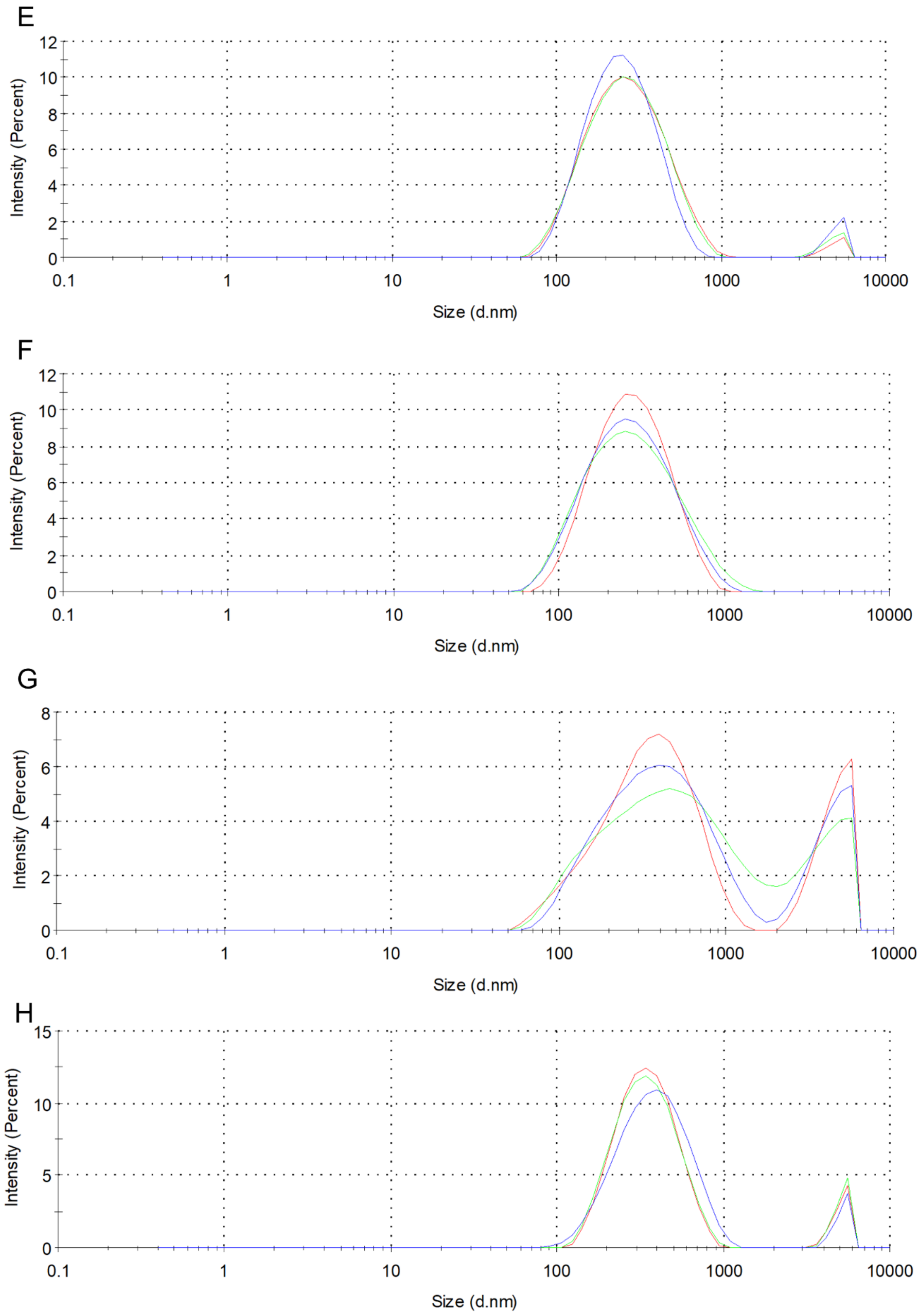

Supplementary Figure S3. Size distribution by intensity obtained from dynamic light scattering analysis (DLS) for CS-TPP-dsRNA and PEI-dsRNA nanoparticles. **A**) CS:TPP:dsRNA (1:1:1). **B**) CS:TPP:dsRNA (5:1:1). **C**) CS:TPP:dsRNA (10:1:1). **D**) PEI:dsRNA (N:P= 1:1). **E**) PEI:dsRNA (N:P= 3:1). **F**) PEI:dsRNA (N:P= 6:1). **G**) PEI:dsRNA (N:P= 10:1). **H**) PEI:dsRNA (N:P= 20:1).

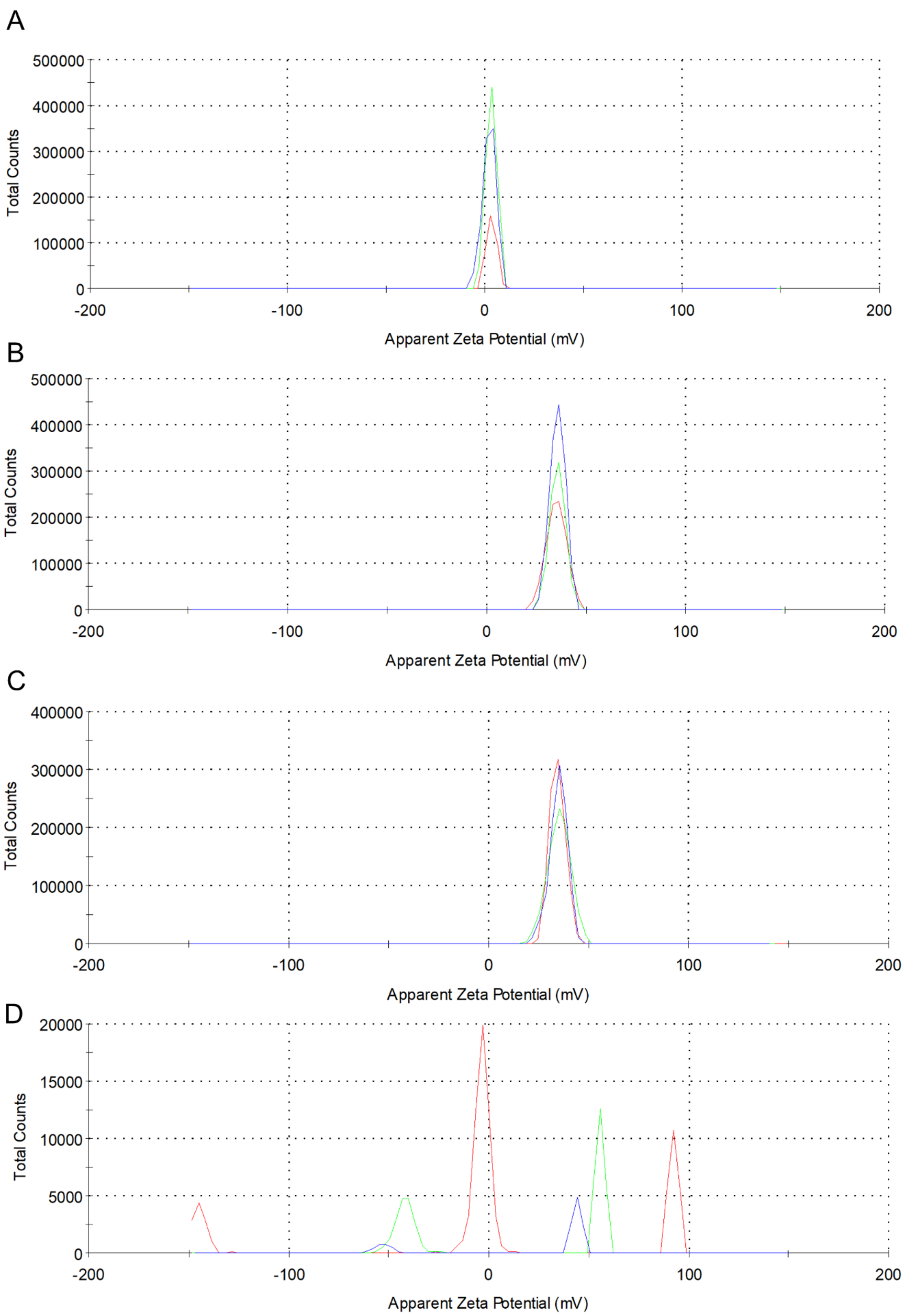
A
B
C
D
Total Counts
Apparent Zeta Potential (mV)
500000
400000
300000
200000
100000
20000
15000
10000
5000
0
-200
-100
100
200

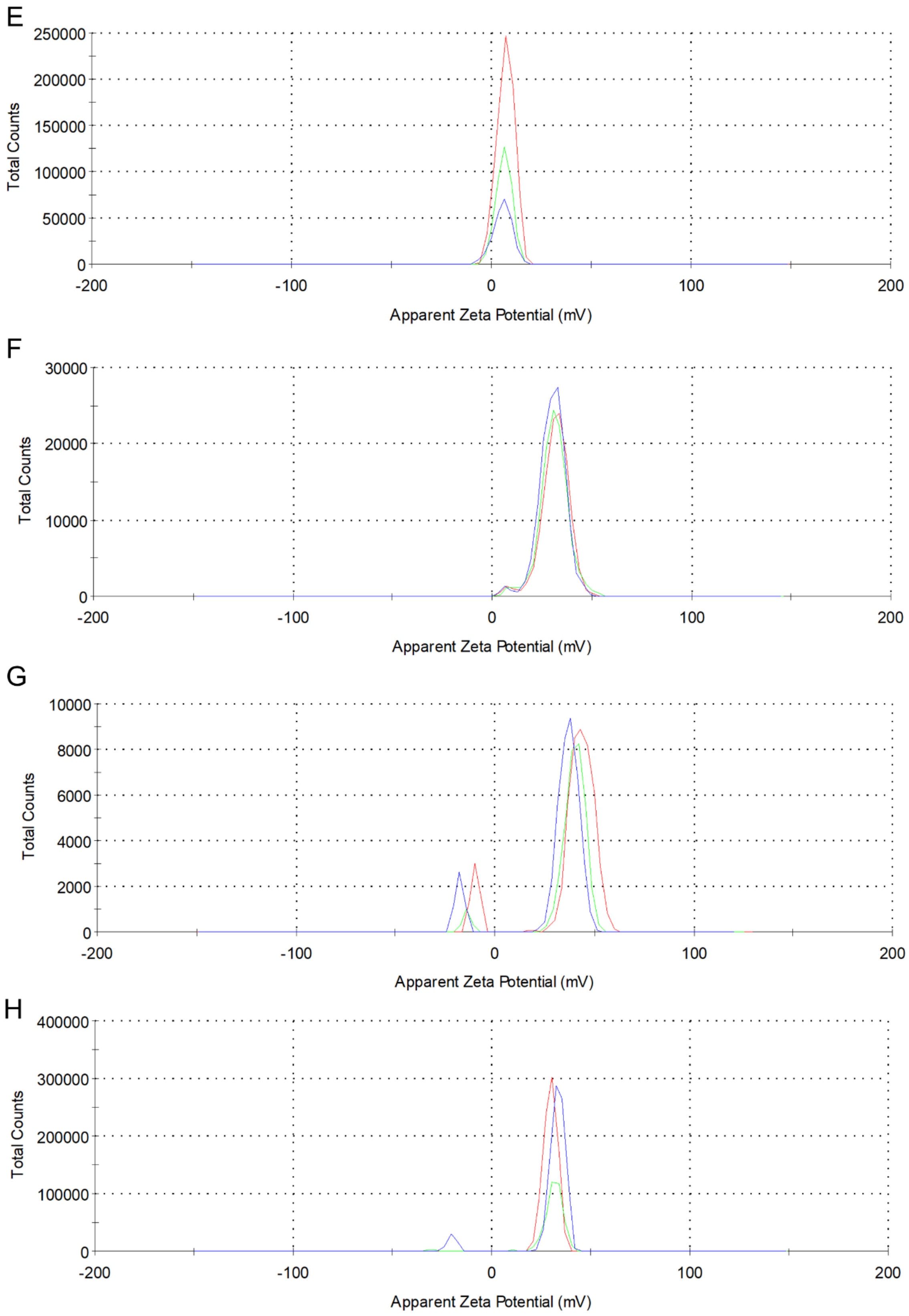

Supplementary Figure S3. Zeta potential profile obtained form dynamic light scattering analysis (DLS) for CS-TPP-dsRNA and PEI-dsRNA nanoparticles. **A**) CS:TPP:dsRNA (1:1:1). **B**) CS:TPP:dsRNA (5:1:1). **C**) CS:TPP:dsRNA (10:1:1). **D**) PEI:dsRNA (N:P= 1:1). **E**) PEI:dsRNA (N:P= 3:1). **F**) PEI:dsRNA (N:P= 6:1). **G**) PEI:dsRNA (N:P= 10:1). **H**) PEI:dsRNA (N:P= 20:1).

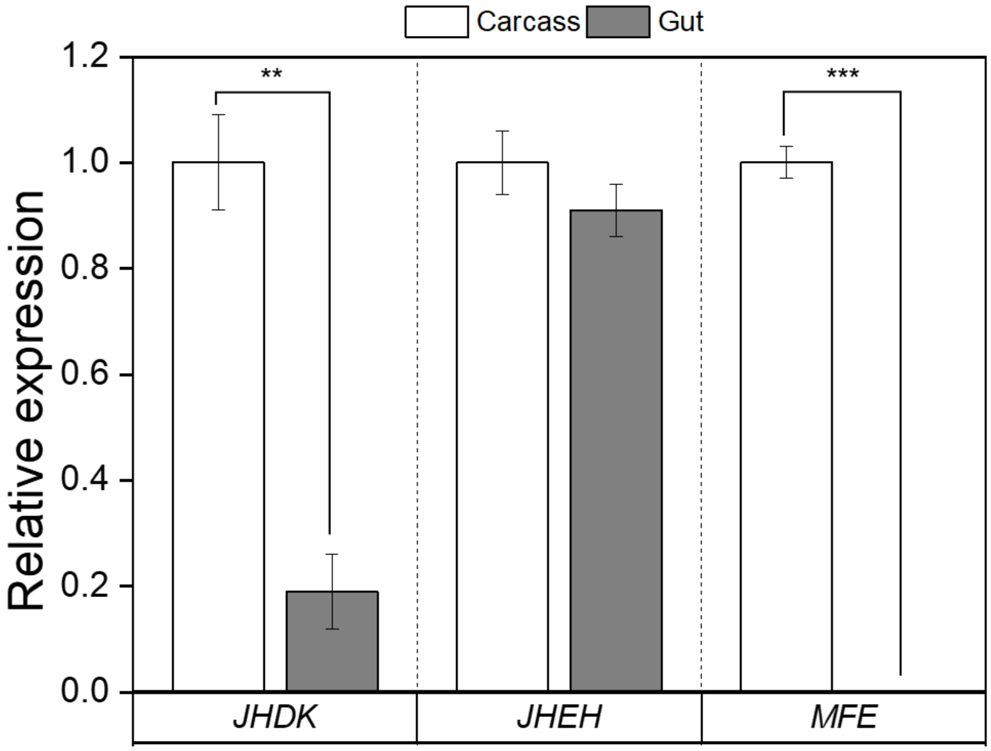

Supplementary Figure S4. Spatial expression profiles of target genes in CBW's third-instar larvae. Relative expression is given as $2^{-\Delta\Delta Ct}$ (fold-change values). The sample with the highest expression was scaled to 1. Asterisks indicate significant differences between tissues at different p-value levels: * < 0.05; ** < 0.01; *** < 0.001 (t-test, Bonferroni correction). *MFE*: *methyl farnesoate epoxidase*. *JHEH*: *juvenile hormone epoxide hydrolase I*. *JHDK*: *juvenile hormone diol kinase*. Carcass: non-digestive system tissue.

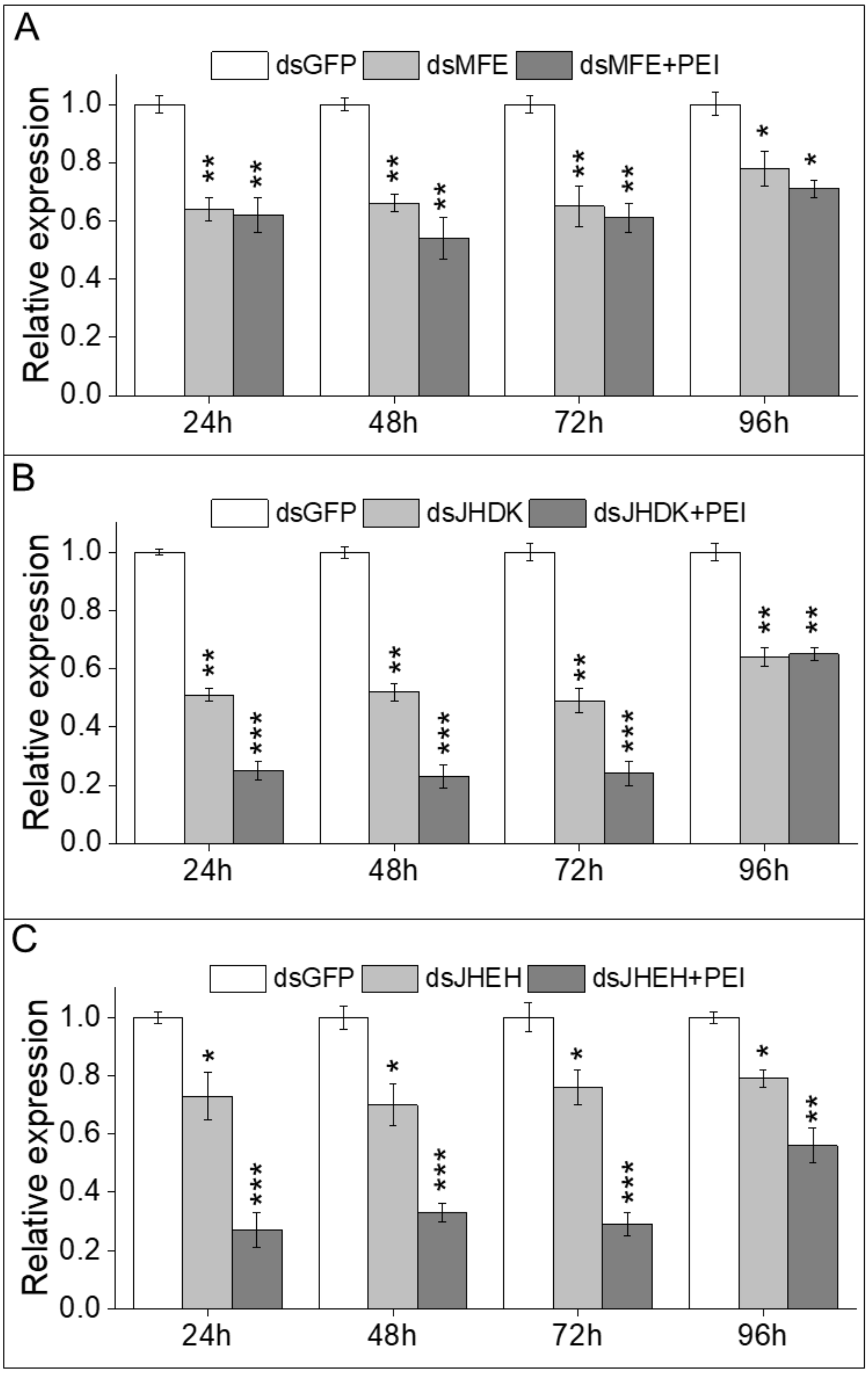

Supplementary Figure S5. Expression of target genes at different time points after injection of naked dsRNA or dsRNA complexed with PEI. **A**) *MFE* expression. **B**) *JHDK* expression. **C**) *JHEH* expression. Relative expression is given as $2^{-\Delta\Delta Ct}$ (fold-change values). The gene expression in ds*GFP* control was scaled to 1. Asterisks indicate significant differences between treatment and control (t-test, bonferroni correction, * $p < 0.05$, ** $p < 0.01$ and *** $p < 0.001$). Data represent mean ± SE (N:12). Third-instar larvae were injected with 0.5 µg of dsRNA. *MFE*: *methyl farnesoate epoxidase*. *JHEH*: *juvenile hormone epoxide hydrolase I*. *JHDK*: *juvenile hormone diol kinase*. *GFP*: *green fluorescent protein gene*.
